# Mutations in *Hcfc1* and *Ronin* result in an inborn error of cobalamin metabolism and ribosomopathy

**DOI:** 10.1038/s41467-021-27759-7

**Published:** 2022-01-10

**Authors:** Tiffany Chern, Annita Achilleos, Xuefei Tong, Matthew C. Hill, Alexander B. Saltzman, Lucas C. Reineke, Arindam Chaudhury, Swapan K. Dasgupta, Yushi Redhead, David Watkins, Joel R. Neilson, Perumal Thiagarajan, Jeremy B. A. Green, Anna Malovannaya, James F. Martin, David S. Rosenblatt, Ross A. Poché

**Affiliations:** 1 Department of Molecular Physiology and Biophysics, Baylor College of Medicine, Houston, TX 77030 USA; 2 Graduate Program in Molecular Physiology and Biophysics, Baylor College of Medicine, Houston, TX 77030 USA; 3 Department of Basic and Clinical Sciences, University of Nicosia Medical School, Nicosia, Cyprus; 4 Graduate Program in Developmental Biology, Baylor College of Medicine, Houston, TX 77030 USA; 5 Department of Biochemistry and Molecular Biology, Baylor College of Medicine, Houston, TX 77030 USA; 6 Department of Pathology, Center for Translational Research on Inflammatory Diseases, Michael E. DeBakey Veterans Affairs Medical Center, Houston, TX 77030 USA; 7 The Francis Crick Institute, London, NW1 1AT UK; 8 Centre for Craniofacial Biology and Regeneration, King’s College London, London, SE1 9RT UK; 9 Division of Medical Genetics, Department of Specialized Medicine, McGill University Health Centre, Montreal, QC Canada; 10 Division of Medical Biochemistry, Department of Specialized Medicine, McGill University Health Centre, Montreal, QC Canada; 11 Development, Disease Models and Therapeutics Graduate Program, Baylor College of Medicine, Houston, TX 77030 USA; 12 Department of Medicine, Baylor College of Medicine, Houston, TX 77030 USA; 13 Texas Heart Institute, Houston, TX 77030 USA; 14 Department of Human Genetics, McGill University, Montreal, QC Canada

**Keywords:** Disease model, Gene expression, Ribosome

## Abstract

Combined methylmalonic acidemia and homocystinuria *(cblC)* is the most common inborn error of intracellular cobalamin metabolism and due to mutations in *Methylmalonic Aciduria type C and Homocystinuria (MMACHC)*. Recently, mutations in the transcriptional regulators *HCFC1* and *RONIN (THAP11)* were shown to result in cellular phenocopies of *cblC*. Since HCFC1/RONIN jointly regulate *MMACHC*, patients with mutations in these factors suffer from reduced *MMACHC* expression and exhibit a *cblC*-like disease. However, additional de-regulated genes and the resulting pathophysiology is unknown. Therefore, we have generated mouse models of this disease. In addition to exhibiting loss of *Mmachc*, metabolic perturbations, and developmental defects previously observed in *cbl*C, we uncovered reduced expression of target genes that encode ribosome protein subunits. We also identified specific phenotypes that we ascribe to deregulation of ribosome biogenesis impacting normal translation during development. These findings identify HCFC1/RONIN as transcriptional regulators of ribosome biogenesis during development and their mutation results in complex syndromes exhibiting aspects of both *cblC* and ribosomopathies.

## Abstract (web summary)

Combined methylmalonic acidemia (MMA) and hyperhomocysteinemias are inborn errors of vitamin B12 metabolism, and mutations in the transcriptional regulators HCFC1 and RONIN (THAP11) underlie some forms of these disorders. Here the authors generated mouse models of a human syndrome due to mutations in RONIN (THAP11) and HCFC1, and show that this syndrome is both an inborn error of vitamin B12 metabolism and displays some features of ribosomopathy.

## Introduction

Cobalamin (vitamin B_12_) is a dietary nutrient essential for normal human development and health. Specifically, methylcobalamin (MeCbl) and adenosylcobalamin (AdoCbl) serve as coenzymes for methionine synthase (MTR) and methylmalonyl-CoA mutase (MUT), respectively^1,2^. MTR is a cytoplasmic enzyme that catalyzes the methylation of homocysteine (Hcy) to generate methionine, an essential amino acid^3^. MUT is localized to the mitochondria where it converts methylmalonyl-CoA to succinyl-CoA, an important intermediate of the citric acid cycle (TCA cycle). The availability of MeCbl and AdoCbl to MTR and MUT is dependent on several intracellular trafficking proteins and enzymes, which effect the conversion of cobalamin intermediates into these necessary coenzymes^4^. Mutations in the genes encoding the proteins responsible for this process comprise eight genetic complementation groups, all of which result in human inborn errors of cobalamin metabolism^5^. The most common of these diseases is combined methylmalonic acidemia and homocystinuria, *cblC* type (OMIM #277400)^6–8^. Patients with *cblC* suffer from a multisystem disease that includes, but is not limited to, intrauterine growth restriction, hydrocephalus, severe cognitive impairment, intractable epilepsy, retinal degeneration, anemia, and congenital heart malformations^9,10^.

Previously, *cblC* was shown to be due to homozygous recessive or compound heterozygous mutations in the gene *Methylmalonic Aciduria type C and Homocystinuria (MMACHC)*^11^. *MMACHC* encodes a protein that traffics cobalamin after it exits the lysosome and also removes the upper ligand as a first step in the conversion of cobalamin to MeCbl and AdoCbl^12–16^. As a consequence, mutations in *MMACHC* lead to reduced levels of both of these cofactors, which in turn result in reduced MTR and MUT activity. The end result is a toxic buildup of the upstream metabolites homocysteine and methylmalonic acid (MMA), and a decrease in methionine^11^. Several studies suggest that homocysteine is an endogenous agonist of the *N*-methyl-D-aspartate (NMDA) receptor, and thus its increase in the above context is thought to result in neuron excitotoxicity and oxidative injury, which both significantly contribute to the central nervous system (CNS) pathology^17–19^. However, the respective contribution of each of these metabolic perturbations to the full pathophysiology of *cblC* is not well-defined.

To add further complexity to the spectrum of cobalamin diseases, an X-linked clinical and cellular variant of *cblC* was recently described and named *cblX* (OMIM #309541)^20^. Rather than being due to mutations in *MMACHC*, this disorder was shown to be due to point mutations in the gene encoding the transcriptional cofactor Host Cell Factor C1 (HCFC1)^20^. The name *cblX* was given to this syndrome to emphasize that the disease is X-linked and requires different genetic counseling from the other disorders of cobalamin metabolism. However, cells from patients with this disorder do not correct cells from *cblC* in somatic cell complementation studies and are thus not a new complementation group, but rather cellular phenocopies of *cblC*. Another patient having a *cblX*-like syndrome carried mutations in the gene encoding the transcription factor THAP Domain Containing 11 (THAP11—also known as RONIN), which was initially identified as an embryonic stem cell (ESC) pluripotency factor^21,22^. Within this patient, the disease was shown to be inherited as an autosomal recessive condition. HCFC1 is an obligate cofactor of RONIN, and previous studies showed that this complex directly regulates the transcription of *MMACHC*^20–24^. Thus, patients with mutations in both *HCFC1* and *RONIN* presumably suffer from reduction of *MMACHC* mRNA levels during development and therefore develop a *cblC*-like disease in utero. However, given the known roles of the RONIN/HCFC1 complex in broadly regulating gene expression, it is very likely that other target genes contribute to the full mutant phenotypes. In fact, it was reported that the neurological features observed in patients with HCFC1 mutations are more severe than what is typically observed in *cblC*, suggesting that MMACHC deficiency alone cannot explain all clinical manifestations^20^.

To gain greater insight into the pathophysiology of these disorders, we generated mouse models carrying the same point mutations as the human patients (*Hcfc1*^*A115V/Y*^ and *Ronin*^*F80L/F80L*^). Phenotyping showed that both lines indeed exhibit combined methylmalonic acidemia and homocystinuria and we observed CNS, hematological, and cardiac defects consistent with *cblC* patients. However, we also observed additional unexpected phenotypes such as craniofacial dysmorphia, homeotic transformations, and white belly spotting, which are frequently observed in ribosomopathies of zebrafish, mice, and humans^25–33^. We performed RNA-seq on embryonic *Ronin*^*F80L/F80L*^ brains along with RONIN ChIP-seq and found that RONIN directly promotes the transcription of a large cohort of genes involved in ribosome biogenesis many of which are cytoplasmic and mitochondrial ribosomal protein subunits. Paradoxically, assessment of global protein translation showed an increase in *Ronin*^*F80L/F80L*^ tissue. However, we also noted a global increase in protein ubiquitination suggesting that this apparent increase in translation may result in aberrant proteins that must be degraded to avoid proteotoxic stress. Interestingly, global mass spectrometry analysis indicated that, among the upregulated proteins, those involved in ribosome biogenesis were enriched. These data are consistent with an unexplained phenomenon that was recently described in budding yeast ribosome protein mutants^34^. It was shown that, depending on whether a large or small subunit protein was deleted, the cell experiences an increase in proteins involved in proteasome-mediated degradation or ribosome biogenesis.

Taken together, this study identifies RONIN and HCFC1 as essential regulators of ribosome biogenesis during embryonic development. We also establish that, in addition to a bona fide cobalamin deficiency syndrome, these transcription factor disorders are also ribosomopathies impacting embryonic development the nature of which is complex and distinct from other known syndromes involving disrupted ribosome biogenesis.

## Results

### Generation of *Hcfc1* and *Ronin* point mutant mouse models

**Fig. 1:**
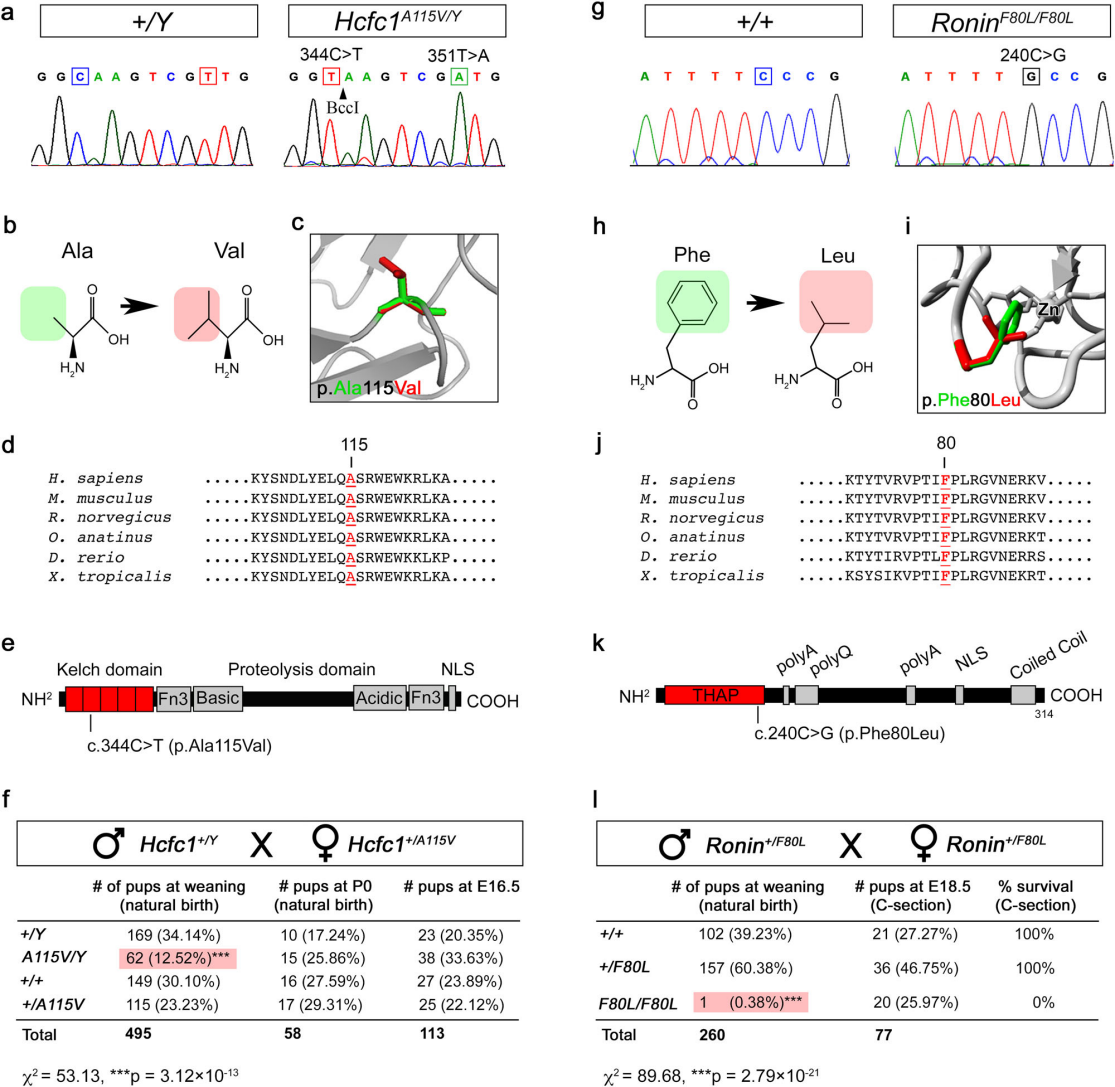
Generation of *Hcfc1* and *Ronin* point mutants. **a** Chromatograph traces of wild type and *Hcfc1 A115V* hemizygous male mice showing the c.344 C>T (p.Ala115Val) mutation and a silent mutation (c.351 T>A) creating a BccI restriction enzyme site. **b** Amino acid structural change due to A115V. **c** Image of the predicted A115V substitution within the HCFC1 protein. **d** Alanine 115 in HCFC1 is evolutionarily conserved through vertebrates. **e** Mouse HCFC1 structure showing the localization of the A115V mutation. **f** Ratios of *Hcfc1*^*A115V/Y*^ male progeny at E16.5^,^ P0, and weaning. Statistically significant differences in the observed genotypes were determined using the chi-squared test (*χ*^2^ = 53.13, ***p = 3.12 × 10^−13^). **g** Chromatograph traces of wild type and *Ronin F80L* homozygous mice showing the c.240 C>G (p.Phe80Leu) mutation. **h** Amino acid structural change due to F80L. **i** Image of the predicted F80L substitution within the RONIN protein. **j** Phenylalanine 80 in RONIN is evolutionarily conserved through vertebrates. **k** Mouse RONIN structure showing the localization of the F80L mutation. **l** Ratios of *Ronin*^*F80L/F80L*^ progeny at E18.5 (C-sectioned) and weaning. Statistically significant differences in the observed genotypes at weaning were determined using the chi-squared test (*χ*^2^ = 89.68, ***p = 2.79 × 10^−21^).

CRISPR/Cas9 genome editing was used to make targeted point mutations in *Hcfc1* and *Ronin* (Fig. 1). The *Hcfc1* c.344 C>T (p.A115V) allele is an alanine to valine substitution at amino acid position 115, which is a highly conserved residue among vertebrates (Fig. 1a–d). Furthermore, this mutation is currently the most commonly observed among patients and maps to the Kelch domain of HCFC1, which is predicted to be a protein-protein interaction domain (Fig. 1e)^20^. To aid in genotyping, we also made a silent mutation (c.351 T>A) that generates a BccI restriction enzyme site (Fig. 1a). Putative *Hcfc1*^*+/A115V*^ founder females were identified by sequencing and crossed to wild type C57BL/6J males to generate F1s. The F1 *Hcfc1*^*+/A115V*^ females were genotyped by PCR and sequencing, and correctly targeted progeny identified for further characterization. These *Hcfc1*^*+/A115V*^ females were crossed to wild type males on the same C57BL/6J genetic background, and we found that *Hcfc1*^*A115V/Y*^ hemizygous male progeny were underrepresented by ~50% at weaning (Fig. 1f). Next, we performed timed matings and found that E16.5 and P0 *Hcfc1*^*A115V/Y*^ males were recovered at Mendelian ratios, which suggests that *Hcfc1*^*A115V/Y*^ mice are sub-viable with half dying postnatally prior to weaning (Fig. 1f).

Due to reports of a patient with homozygous point mutations in *RONIN* and a disorder similar to those with mutations in *HCFC1*, we made an additional mouse model with the same mutation. The *Ronin* c.240 C>G (p.F80L) allele is a phenylalanine to leucine substitution at amino acid position 80, which is a conserved residue in the THAP DNA binding domain of RONIN (Fig. 1g, k)^21^. Putative *Ronin*^*+/F80L*^ founders, on a C57BL/6J background, were identified by screening with the Surveyor® Mutation Detection Kit (Integrated DNA Technologies). Mice with apparent mismatches were selected and crossed to wild type C57BL/6J mice to generate F1s and the F1 progeny were sequenced to identify correctly targeted *Ronin*^*+/F80L*^ mice. Confirmed *Ronin*^*+/F80L*^ mice were intercrossed, and their progeny genotyped. Out of 260 mice, we recovered a single *Ronin*^*F80L/F80L*^ weanling (Fig. 1l). This escaper lived to approximately one month of age and was runted as compared to littermates. The failure to recover additional *Ronin*^*F80L/F80L*^ mice suggested that the majority die prior to weaning. We next performed Caesarian sections on pregnant dams and found a Mendelian ratio of E18.5 *Ronin*^*F80L/F80L*^ mice. As indicated by gasping motions, the *Ronin*^*F80L/F80L*^ pups were alive, but appeared cyanotic and 0/20 survived past approximately 30 min (Supplementary Fig. 1a and Supplementary Movie 1). Based on the additional finding that lungs isolated from these mutants failed to float in water, we conclude that lethality is due to an inability to breathe (Supplementary Fig. 1b). The fact that the *Ronin*^*F80L/F80L*^ pups exhibited the drive to breathe, yet could not inflate their lungs with air, argued against a brain stem defect. This conclusion was further supported by the finding that *Krox20-Cre* and *Phox2b-Cre* mediated brain stem conditional knockouts (CKOs) of *Ronin* are viable at weaning (Supplementary Fig. 1c)^35,36^. However, lung histology, and diaphragm positioning and innervation appeared grossly normal, possibly suggesting another origin of *Ronin*^*F80L/F80L*^ neonatal inability to breathe (Supplementary Fig. 1d–f).

We next determined the consequence of the *Hcfc1 A115V* and *Ronin F80L* mutations on RNA and protein expression. Quantitative rtPCR (qrtPCR) and western blot analysis of E16.5 *Hcfc1*^*A115V/Y*^ brains showed that the A115V mutation alters neither *Hcfc1* mRNA nor protein levels (Supplementary Fig. 2a, b). In contrast, *Ronin*^*F80L/F80L*^ brains showed a significant increase in *Ronin* transcripts suggesting that RONIN may be a transcriptional repressor of itself (Supplementary Fig. 2c). This interpretation is consistent with our published RONIN chromatin immunoprecipitation sequencing (ChIP-seq) experiments showing RONIN binds to its own promoter^23^. Western blot showed that the RONIN F80L protein levels were significantly reduced, and a cycloheximide chase experiment determined that this is likely due to mutant protein instability (Supplementary Fig. 2d–f). Despite the mutations in *Hcfc1* or *Ronin*, both proteins were able to be co-immunoprecipitated (Co-IP) from embryonic brain lysates suggesting that in the context of both single mutations, the RONIN/HCFC1 protein complex still forms but is not fully functional (Supplementary Fig. 2g, h).

### *Hcfc1*^*A115V/Y*^ and *Ronin*^*F80L/F80L*^ mice exhibit *cblC*-like metabolic perturbations

**Fig. 2:**
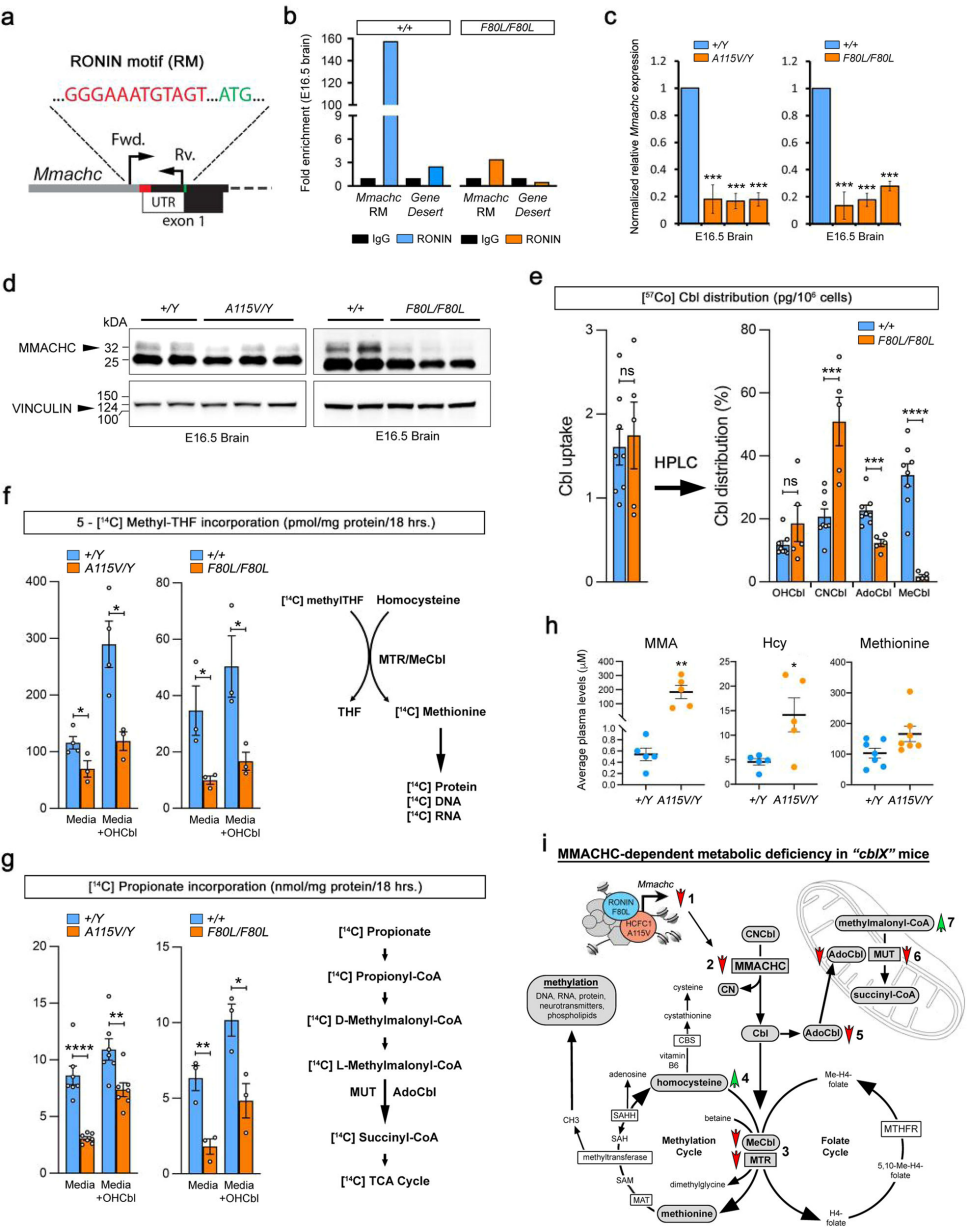
Cobalamin-related metabolic perturbations in *Hcfc1*^*A115V/Y*^ and *Ronin*^*F80L/F80L*^ mice. **a** RONIN binding motif within the *Mmachc* promoter. **b** RONIN ChIP-PCR of the *Mmachc* promoter. **c** QrtPCR analysis (*n* = 3 per genotype) of *Mmachc* in the *Hcfc1*^*A115V/Y*^ and *Ronin*^*F80L/F80L*^ embryonic brains. **d** Western blot analysis of MMACHC in the *Hcfc1*^*A115V/Y*^ and *Ronin*^*F80L/F80L*^ embryonic brains. **e** [^57^Co] cyanocobalamin incorporation assay (*n* = 8 wild type and *n* = 5 mutants) to determine cobalamin distribution in MEFs. **f** 5-[^14^C] methyltetrahydrofolate (methylTHF) incorporation assay of methionine synthase (MTR) activity in MEFs (*n* = 3 or 4 wild type and *n* = 3 mutants). **g** [^14^C] propionate incorporation assay of methylmalonyl-CoA mutase activity in MEFs (*n* = 3 or 7 wild type and *n* = 3 or 7 mutants). **h** Plasma levels of methylmalonic acid (MMA), homocysteine (Hcy), and methionine (*n* = 5 or 7 wild type and *n* = 5 or 7 mutants). **i** Summary of MMACHC-dependent metabolic deficiency identified in *Ronin* and *Hcfc1*mutant mice. RONIN F80L and HCFC1 A115V mutant protein function is compromised leading to reduced *Mmachc* transcription and protein activity (1 and 2). This defect results in reduced production of MeCbl and reduced MTR activity (3), and the corresponding buildup of the upstream metabolite homocysteine (4). Reduced MMACHC also leads to lower levels of AdoCbl production and MUT activity (5 and 6), and the buildup of methylmalonyl-coA (7). All data are shown as mean ± SEM and *n* ≥ 3 biologically independent samples per genotype. Statistically significant differences between genotypes were determined using the *t* test (two-tailed). *p < 0.05, **p < 0.01, ***p < 0.001, ****p < 0.0001. Source data are provided as a Source data file.

To validate the *Hcfc1*^*A115V/Y*^ and *Ronin*^*F80L/F80L*^ mice as models of *cblC*-like disorders, we assessed them for changes in *Mmachc* expression and the consequential metabolic defects. Our previous retinal ChIP-seq experiment identified a putative RONIN binding motif within the *Mmachc* promoter^23^. ChIP-PCR showed that RONIN binds the same site within the embryonic brain and Luciferase assays further support the conclusion that RONIN drives expression from the *Mmachc* promoter (Fig. 2a, b and Supplementary Fig. 2i, j). Consistent with a previous report, ChIP-PCR showed that the RONIN F80L protein can no longer bind this site (Fig. 2b)^37^.

Importantly, as observed in the patients, *Hcfc1*^*A115V/Y*^ and *Ronin*^*F80L/F80L*^ mice exhibit dramatic reduction in *Mmachc* RNA and protein expression (Fig. 2c, d). These data support the conclusion that RONIN and HCFC1 jointly promote the expression of *Mmachc* during development. To verify that reduction in MMACHC levels translate into a cobalamin deficiency, we derived mouse embryonic fibroblasts (MEFs) from *Ronin*^*F80L/F80L*^ mice and determined that MeCbl and AdoCbl coenzyme forms are greatly reduced (Fig. 2e). *Hcfc1*^*A115V/Y*^ and *Ronin*^*F80L/F80L*^ MEFs also displayed the consequential reduction in the functional activity of MTR and MUT (Fig. 2f, g). Consistent with this result, plasma from adult *Hcfc1*^*A115V/Y*^ mice displayed elevated levels of MMA and Hcy (Fig. 2h). We did not detect a significant decrease in methionine levels (Fig. 2h). In total, these data demonstrate that, due to loss of HCFC1 or RONIN function, and reduced *Mmachc* target gene expression, the *Hcfc1*^*A115V/Y*^ and *Ronin*^*F80L/F80L*^ mice are bona fide models of combined methylmalonic acidemia and homocystinuria (Fig. 2i).

### *Hcfc1*^*A115V/Y*^ and *Ronin*^*F80L/F80L*^ mice exhibit phenotypes observed in *cblC* and patients with mutations in *HCF1* and *RONIN*

**Fig. 3:**
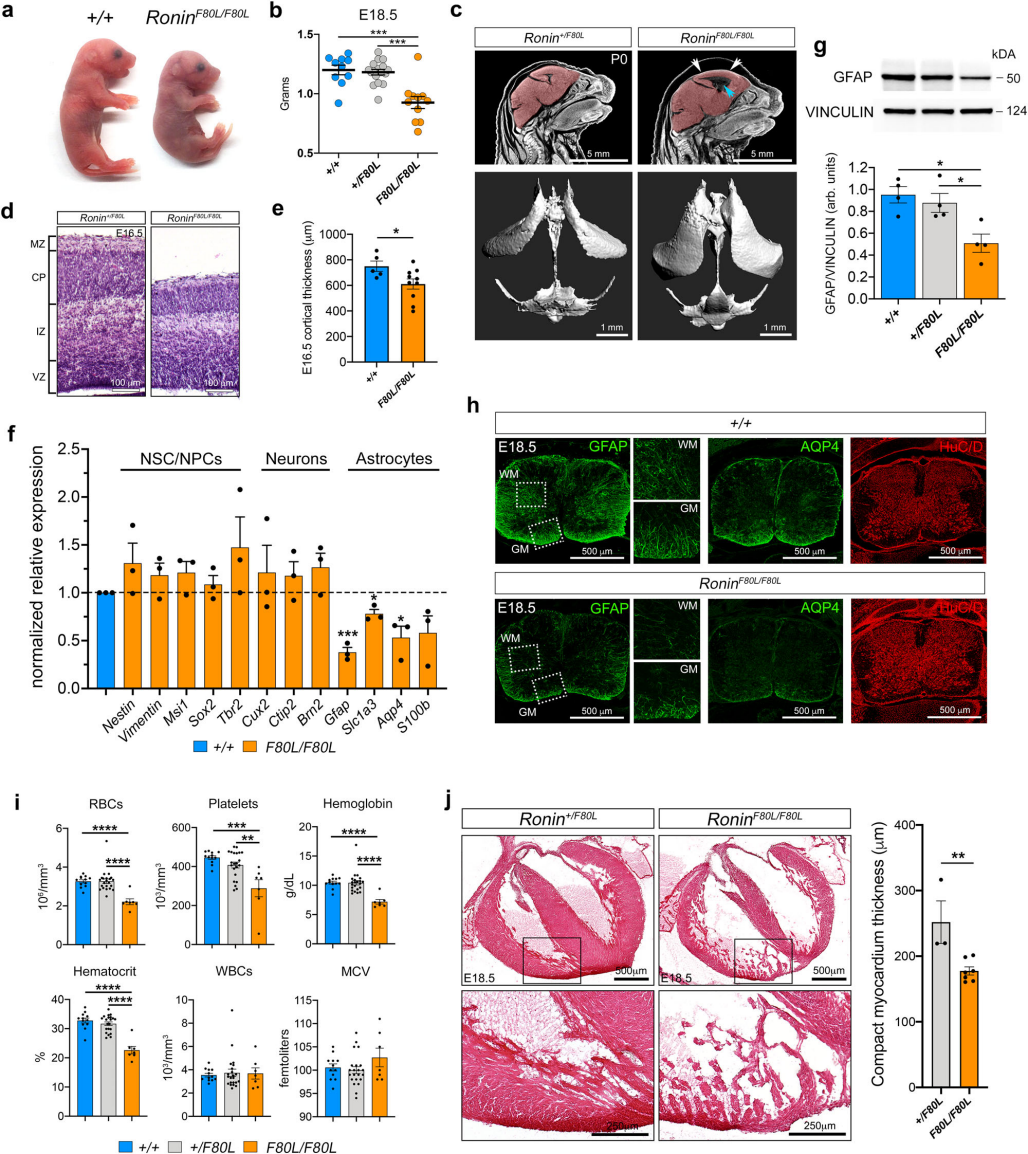
*Ronin*^*F80L/F80L*^ mice exhibit *cblC*-like phenotypes. **a**, **b** Runted E18.5 *Ronin*^*F80L/F80L*^ mice with lower body weights (data are shown as mean ± SEM, *n* = 10 +/+, *n* = 17+/*F80L*, *n* = 11 *F80L/F80L*, statistically significant differences between genotypes were determined using ANOVA and Tukey’s multiple comparisons test). **c** Micro-CT analysis of the CNS showing expanded ventricles (blue arrow) and thinner cortex (white arrows) in *Ronin*^*F80L/F80L*^ neonates. **d**, **e** Hematoxylin and Eosin (H&E) staining showing cortical thinning in *Ronin*^*F80L/F80L*^ mice (data are shown as mean ± SEM, *n* = 5 +/+, *n* = 10 *F80L/F80L*, statistically significant differences between genotypes were determined using the *t* test, *p < 0.05). **f** E18.5 brain qrtPCR analysis (data are shown as mean ± SEM, *n* = 3 per genotype, statistically significant differences between genotypes were determined using the *t* test (two-*t*ailed), *p < 0.05, ***p < 0.001). **g** E18.5 brain western blot analysis (data are shown as mean ± SEM, *n* = 4 per genotype, statistically significant differences between genotypes were determined using ANOVA and Tukey’s multiple comparisons test, *p < 0.05). **h** E18.5 spinal cord immunofluorescence. **i** Peripheral blood analysis (data are shown as mean ± SEM, *n* = 12 + /+, *n* = 22+*/F80L*, *n* = 7 *F80L/F80L*, statistically significant differences between genotypes were determined using ANOVA and Tukey’s multiple comparisons test, **p < 0.01, ***p < 0.001, ****p < 0.0001). **j** H&E staining showing hypertrabeculation and ventricular wall thinning of the *Ronin*^*F80L/F80L*^ heart. Data are shown as mean ± SEM, *n* = 3+/F*80L*, *n* = 7 *F80L/F80L*, statistically significant differences between genotypes were determined using the *t* test (two-tailed). **p < 0.01. Source data are provided as a Source data file.

Based on the observed disruptions in cobalamin metabolism, we next sought to determine the consequence of the *Hcfc1 A115V* and *Ronin F80L* mutations on embryonic development. Consistent with the intrauterine growth restriction seen in the human *cblC* patients, *Hcfc1*^*A115V/Y*^ and *Ronin*^*F80L/F80L*^ mice are slightly runted during embryogenesis and the surviving *Hcfc1*^*A115V/Y*^ mice remain so postnatally (Fig. 3a, b and Supplementary Fig. 3a)^9^. Since the *Ronin*^*F80L/F80L*^ mice die at birth, developmental defects were expected. Indeed, *Ronin*^*F80L/F80L*^ brains exhibit thinning of the cerebral cortex and marked expansion of the lateral ventricles, which suggested a possible defect in neurogenesis (Fig. 3c–e). However, qrtPCR, immunofluorescence, EdU/Caspase 3 labeling, and western blot analysis of E18.5 mutant brains showed no obvious differences in the neural stem cell (NSC), progenitor cell (NPC), or postmitotic neuron populations (Fig. 3f). Surprisingly, we noticed a significant reduction in astrocyte-specific transcripts *Gfap, Slc1a3, Aqp4*, and *S100b* and GFAP reduction within the brain was confirmed at the protein level (Fig. 3f, g). These data suggested that the *Ronin*^*F80L/F80L*^ embryonic brain may suffer from a deficiency in astrogliogenesis. Consistent with this idea, we generated E16.5 *Ronin*^*F80L/F80L*^ brain RNA-seq data and found that expression of the proneural gene *Ascl1*, and the anti-astrogliogenic transcription factor *Emx2* and DNA methyltransferase *Dnmt1* were also significantly increased (Supplementary Dataset 1)^38–41^. Since gliogenesis in the mouse cortex begins midgestation, it remained possible that the reduction of astrocyte markers at E18.5 is due to a general developmental delay. Therefore, we analyzed E18.5 spinal cords, which begin gliogenesis at E12.5^42^. Immunofluorescence for neuronal marker HuC/D showed no apparent change between wild type and *Ronin*^*F80L/F80L*^ mice (Fig. 3h). However, differentiated astrocyte markers GFAP and AQP4 showed an obvious qualitative reduction in the mutants in both white and gray matter (Fig. 3h). In total, these data suggest that disrupted astrogliogenesis may underlie the CNS pathophysiology of patients with *HCFC1* and *RONIN* mutations.

Other potential causes of lethality might be defects in the hematopoietic or cardiovascular systems, which have both been associated with *cblC*^9^. Analysis of *Ronin*^*F80L/F80L*^ blood at E18.5, prior to death at P0, revealed significant reduction in red blood cells (RBCs), platelets, hemoglobin, and hematocrit. However, white blood cell (WBC) numbers and the mean corpuscular volume (MCV) were not significantly altered (Fig. 3i). Micro-CT and histology of E18.5 hearts revealed that *Ronin*^*F80L/F80L*^ mice suffer from ventricular hypertrabeculation and thinning of the myocardium (Fig. 3j).

Since only half of the *Hcfc1*^*A115V/Y*^ mice die postnatally, whereas all of the *Ronin*^*F80L/F80L*^ mice die at P0, we expected to observe greater variability in *Hcfc1*^*A115V/Y*^ phenotype severity.

Indeed, among the *Hcfc1*^*A115V/Y*^ mice analyzed at E18.5, a subset exhibited thinning of the cerebral cortex and ventricular myocardium similar to the *Ronin*^*F80L/F80L*^ mice whereas others were indistinguishable from wild type (Supplementary Fig. 3b–j). On average, we did not detect significant changes in the hematopoietic system, but a subset of mutants showed reduced RBC numbers, hematocrits, and hemoglobin levels (Supplementary Fig. 3k).

### *Hcfc1*^*A115V/Y*^ and *Ronin*^*F80L/F80L*^ mice display unexpected craniofacial phenotypes

**Fig. 4:**
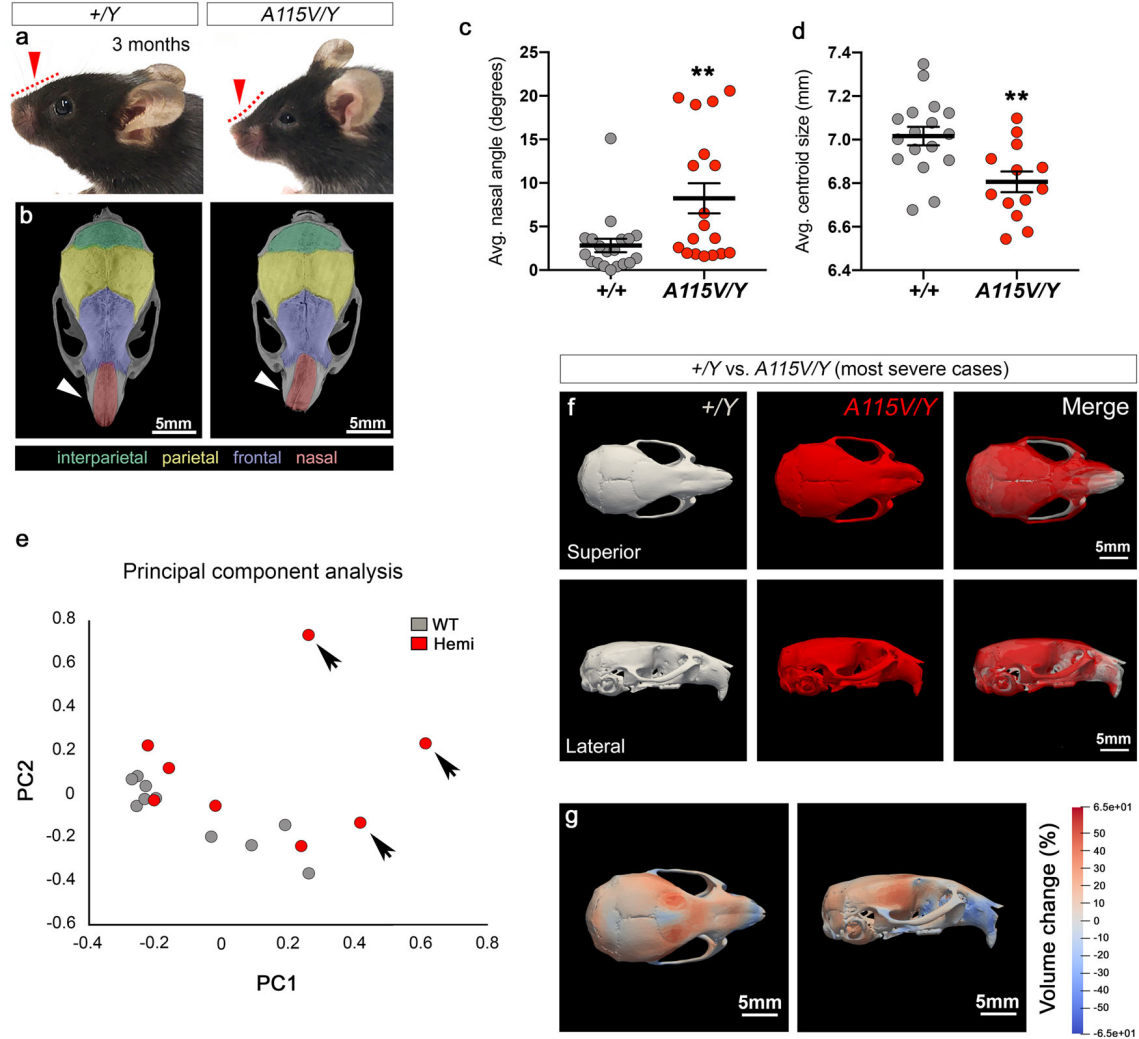
Craniofacial dysmorphia in adult *Hcfc1*^*A115V/Y*^ mice. **a**, **b** *Hcfc1*^*A115V/Y*^ mice exhibit a tilted nasal angle (red arrows and dashed lines) and a curved snout (white arrowheads). **c** A subset of the *Hcfc1*^*A115V/Y*^ mice showed a nasal angle over 10°. Data are shown as mean ± SEM, *n* = 19 wild type, *n* = 18 mutants, statistically significant differences between genotypes were determined using the *t* test (two-tailed). **p < 0.006. **d** *Hcfc1*^*A115V/Y*^ mice have a smaller centroid size than wild type. Data are shown as mean ± SEM, *n* = 17 wild type, *n* = 13 mutants, statistically significant differences between genotypes were determined using the *t* test (two-*t*ailed). **p = 0.003. **e** PCA of the landmark-free morphometrics showing a subset of *Hcfc1*^*A115V/Y*^ mice with a significant deviation from wild type (arrows). **f** Superior and lateral views of the averaged skull shape of the three most severely affected *Hcfc1*^*A115V/Y*^ mice overlaid with the average of three wild type^45^. **g** Stretch heatmap showing the % volume change between the three most severely affected *Hcfc1*^*A115V/Y*^ mice compared to controls. Source data are provided as a Source data file.

Over the course of phenotyping the *Hcfc1*^*A115V/Y*^ and *Ronin*^*F80L/F80L*^ mutants we consistently observed additional features that are not reported in the *cblC* literature. The most penetrant of these defects was craniofacial dysmorphia. As the *Hcfc1*^*A115V/Y*^ mice matured postnatally, we noticed that they exhibited a shortened facial profile and upward curvature of the snout (Fig. 4a, red arrows and lines). Among these mice, a subset also showed pronounced curvature of the nasal bone away from the midline of over 10° (Fig. 4b, arrowheads and c). Interestingly, there is a single report of brothers with *HCFC1* mutations and strikingly similar craniofacial malformations^43^. To further analyze this craniofacial phenotype, superior views of skull micro-CT images were used for two-dimensional quantification of surface area, length, and width of the craniofacial bones as previously described^44^. We determined that the average surface area of the nasal, frontal, and parietal bones are smaller in *Hcfc1*^*A115V/Y*^ mutant mice while no difference shown in interparietal bones. Nasal bone length, nasal bone width, and frontal bone length are shorter in the mutant mice. Frontal bone width, parietal bone length, interparietal bone length and width, and the distance between left and right anterolateral corner of the frontal bone are not changed (see below)^44^. We next performed landmark-free morphometrics as previously described^45^. Quantification of the average centroid size indicated a global size defect in which the mutant skulls are smaller than wild type (Fig. 4d). Principal component analysis (PCA) of the shape changes revealed that the mutant skulls fell into three categories (Fig. 4e). The first group of 2 hemizygous mutants showed no changes in skull shape. The second group of 3 mutants showed a mild midfacial hypoplasia phenotype indicated by a reduction in dimensions of the snout and midface. In the most severe case, 3 mutants had both the midfacial hypoplasia as well as unilateral deviation away from the midline (Fig. 4e, arrows and f). Quantification of the percent volume change between the three most severely affected *Hcfc1*^*A115V/Y*^ mice compared to controls showed that the most significant reduction in volume occurs in the frontal and nasal bones (Fig. 4g).

**Fig. 5:**
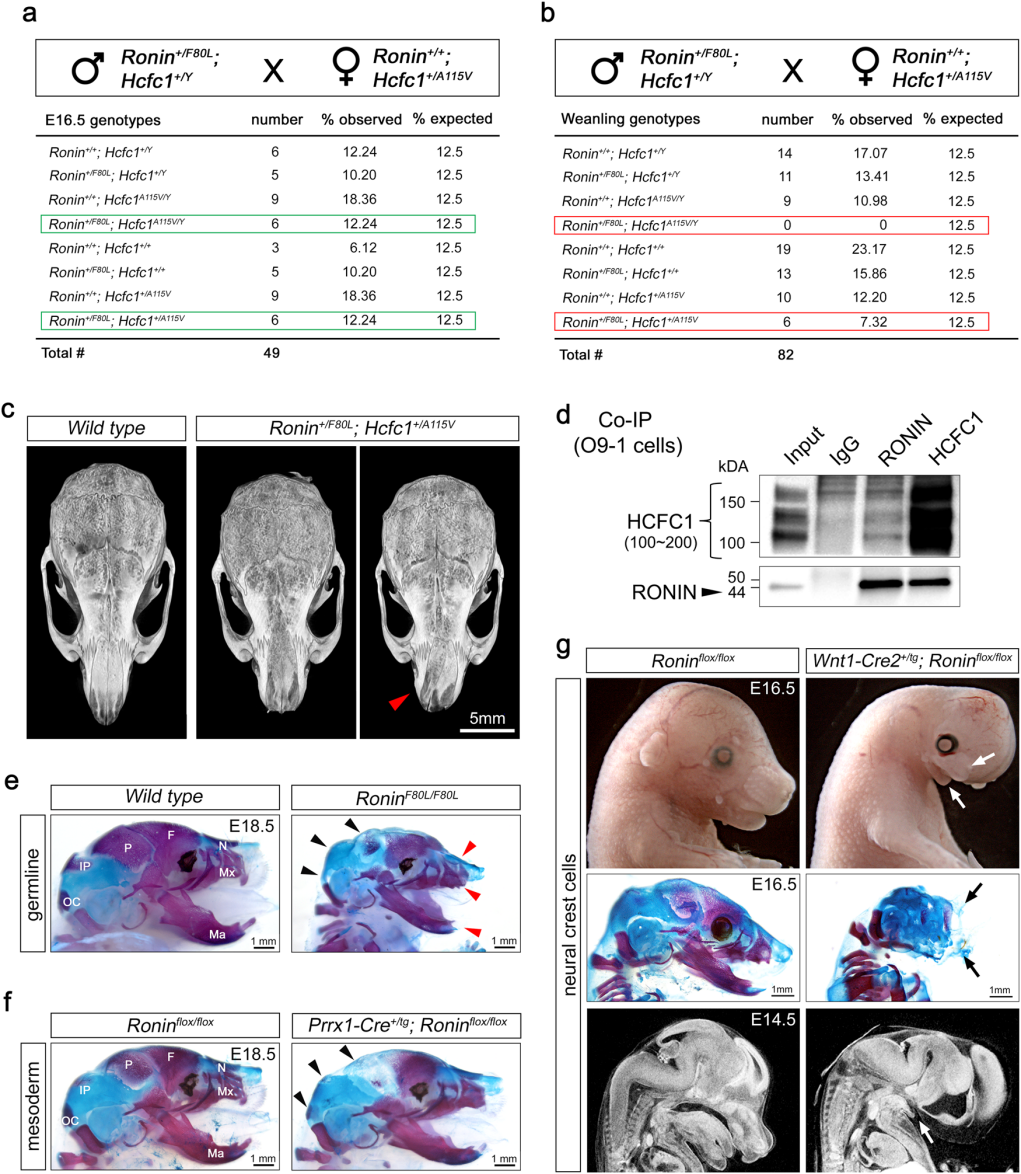
*Ronin* and *Hcfc1* genetically interact to regulate craniofacial development. **a** Crosses revealing the expected Mendelian ratios at E16.5 (green boxes). **b** Crosses revealing underrepresentation of *Ronin/Hcfc1* double heterozygotes and complete lethality of *Ronin*^*+/F80L*^*; Hcfc1*^*A115V/Y*^ by weaning (red boxes). **c** Craniofacial dysmorphia similar to the *Hcfc1*^*A115V/Y*^ mice (red arrowhead). **d** RONIN and HCFC1 co-immunoprecipitation. **e** Skeletal staining showing *Ronin*^*F80L/F80L*^ craniofacial hypoplasia of both mesoderm- (black arrowheads) and neural crest-derived (red arrowheads) bones. **f** Mesoderm-specific *Ronin* CKO (*Prrx1-Cre*^*+/tg*^*; Ronin*^*flox/flox*^) showing loss of mesoderm-derived bones (black arrowheads). **g** Neural crest-specific *Ronin* CKO (*Wnt1-Cre2*^*+/tg*^*; Ronin*^*flox/flox*^) showing agenesis of the neural crest-derived craniofacial skeleton (arrows). For all data (**c**–**g**) *n* = 3 biologically independent samples per genotype. Source data are provided as a Source data file.

**Fig. 6:**
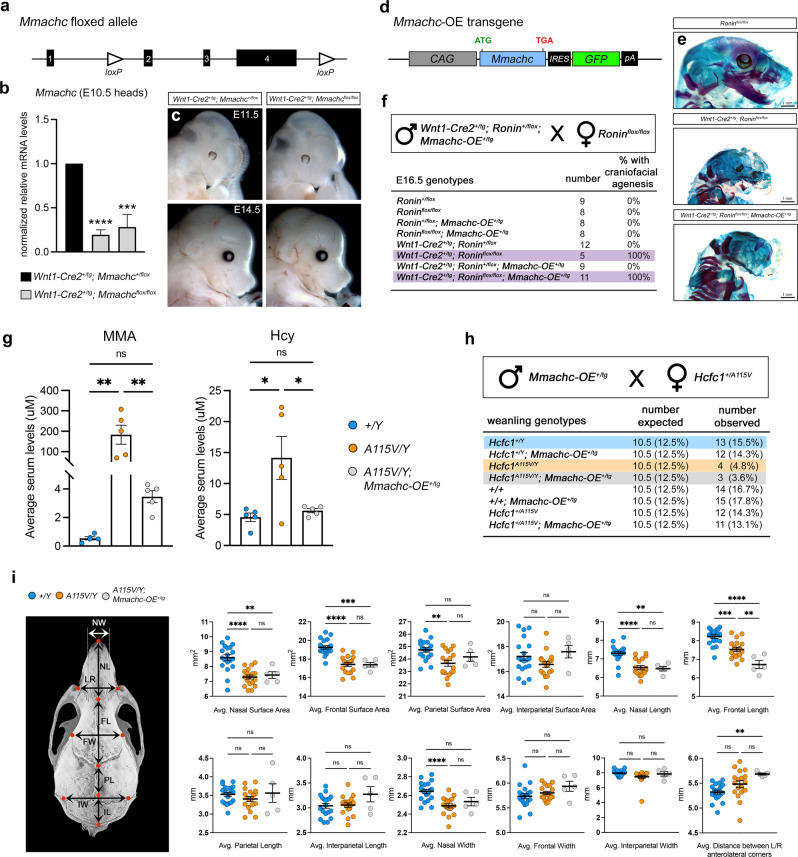
*Mmachc* reduction is not responsible for the *Hcfc1*^*A115V/Y*^ and *Ronin*^*F80L/F80L*^ craniofacial phenotypes. **a** Schematic of the *Mmachc* floxed allele^49^. **b** QrtPCR analysis of E10.5 *Wnt1-Cre2*^*+/tg*^*; Mmachc*^*flox/flox*^ neural crest-specific CKOs. Data are shown as mean ± SEM, *n* = 2 controls, *n* = 2 mutants, statistically significant differences between genotypes were determined using the *t* test (two-tailed). ****p = 2.04 × 10^−6^, ***p = 0.0004. **c** CKO craniofacial development appeared indistinguishable from wild type. **d** Schematic of the *Mmachc* overexpression transgene *(Mmachc-OE*)^49^. **e** Alizarin red and alcian blue-stained skeletons. **f** The *Mmachc-OE* transgene fails to rescue the craniofacial agenesis of *Wnt1-Cre2*^*+/tg*^*; Ronin*^*flox/flox*^ mice. **g** Average serum MMA and Hcy levels (data are shown as mean ± SEM, *n* = 5 biologically independent samples per genotype, statistically significant differences between genotypes were determined using ANOVA and Tukey’s multiple comparisons test, *p < 0.05, **p < 0.01). **h** Pup survival to weaning. **i** Quantified surface area, length, and width of the craniofacial bones (data are shown as mean ± SEM, *n* = 19+/*Y*, *n* = 18 *A115V/Y*, *n* = 5 *A115V/Y; Mmachc-OE* biologically independent samples per genotype, statistically significant differences between genotypes were determined using ANOVA and Tukey’s multiple comparisons test, **p < 0.01, ***p < 0.001, ****p < 0.0001). Abbreviations: nasal bone length (NL), nasal bone width (NW), frontal bone length (FL), frontal bone width (FW), parietal bone length (PL), interparietal bone length (IL), interparietal bone width, (IW) and the distance between left and right anterolateral corner of the frontal bone (LR). Source data are provided as a Source data file.

We next aimed to determine whether the *Ronin F80L* mutation also impacts the craniofacial skeleton. We set up crosses between *Ronin*^*+/F80L*^*; Hcfc1*^*+/Y*^ males and *Ronin*^*+/+*^*; Hcfc1*^*+/A115V*^ females and analyzed the progeny. At E16.5, we recovered both *Ronin*^*+/F80L*^*; Hcfc1*^*+/A115V*^ and *Ronin*^*+/F80L*^*; Hcfc1*^*A115V/Y*^ double mutants at Mendelian ratios (Fig. 5a). However, by weaning, while the *Ronin*^*+/F80L*^*; Hcfc1*^*+/A115V*^ double heterozygous mice were viable, the *Ronin*^*+/F80L*^*; Hcfc1*^*A115V/Y*^ mutants were not (Fig. 5b). This genetic evidence is consistent with the notion that RONIN and HCFC1 functionally interact during critical developmental processes. We also observed that 2 out of 3 *Ronin*^*+/F80L*^*; Hcfc1*^*+/A115V*^ adults exhibited craniofacial dysmorphia similar to the *Hcfc1*^*A115V/Y*^ mice (Fig. 5c), and that RONIN and HCFC1 co-immunoprecipitate (co-IP) from the O9-1 cranial neural crest cell line (Fig. 5d). Together these data support the conclusion that RONIN and HCFC1 cooperatively regulate craniofacial development^46^. It is important to note that E18.5 *Ronin*^*F80L/F80L*^ mice also appeared to have defects in the craniofacial skeleton with a reduction in both the mesoderm and neural crest-derived bones (Fig. 5e, arrowheads). Due to the possibility of developmental delay resulting in this phenotype, we generated CKOs of *Ronin* in both the mesoderm and neural crest craniofacial derivatives. Indeed, *Prrx1-Cre*^*+/tg*^*; Ronin*^*flox/flox*^ CKOs showed a dramatic loss of mesoderm-derived bones (Fig. 5f, arrowheads) whereas *Wnt1-Cre2*^*+/tg*^*; Ronin*^*flox/flox*^ CKOs showed almost complete agenesis of the neural crest-derived bones (Fig. 5g, arrows)^47,48^. In total, these data suggested that mutant craniofacial dysmorphia may be due to target genes other than *Mmachc*. To formally test this idea, we generated an *Mmachc* floxed allele and performed *Wnt1-Cre2* mediated CKO within the neural crest and found that these embryos have no obvious craniofacial phenotype (Fig. 6a–c)^49^. We also generated a transgenic mouse line that overexpresses functional *Mmachc (Mmachc-OE*^*+/tg*^*)* and determined that it was incapable of rescuing the *Wnt1-Cre2*^*+/tg*^*; Ronin*^*flox/flox*^ phenotype (Fig. 6d–f)^49^. The *Mmachc-OE* transgene was also crossed to the *Hcfc1*^*A115V/Y*^ mice, and we determined that the *Hcfc1*^*A115V/Y*^*; Mmachc-OE*^*+/tg*^ offspring have plasma MMA and Hcy levels that are similar to wild type mice thereby suggesting a rescue of the inborn error of cobalamin metabolism (Fig. 6g). However, these mice exhibit the same partial lethality we observed for the *Hcfc1*^*A115V/Y*^ mutants (Fig. 6h). To address possible *Mmachc*-mediated rescue of the *Hcfc1*^*A115V/Y*^ craniofacial dysmorphia, we quantified skull micro-CT images from *Hcfc1*^*A115V/Y*^*; Mmachc-OE*^*+/tg*^ mice and compared these data to wild type and *Hcfc1*^*A115V/Y*^ mice. The craniofacial dysmorphia, especially for the more severely affected frontal and nasal bone surface area and width, did not improve in the *Hcfc1*^*A115V/Y*^*; Mmachc-OE*^*+/tg*^ mice (Fig. 6i). Taken together, these data implicated an *Mmachc*-independent component of *Hcfc1*^*A115V/Y*^ and *Ronin*^*F80L/F80L*^ developmental disorders, which motivated us to identify additional RONIN/HCFC1 target genes.

### RONIN and HCFC1 regulate a large cohort of genes essential for ribosome biogenesis

We isolated total mRNA from E16.5 *Ronin*^*F80L/F80L*^ and wild type brains and performed RNA-seq. Differential expression analysis revealed 980 genes that were upregulated and 1,301 that were downregulated in *Ronin*^*F80L/F80L*^ brains (Supplementary Fig. 4 and Supplementary Dataset 1). Gene ontology (GO) analysis showed that genes encoding transcription factors, cell cycle regulators, and brain development were upregulated (Supplementary Fig. 4b and Supplementary Dataset 1). Among the downregulated genes, we found that structural constituents of the ribosome were enriched (Supplementary Fig. 4c), including approximately half of the genes that encode both cytoplasmic and mitochondrial ribosome protein subunits (Supplementary Fig. 5a, b and Supplementary Dataset 1). The next most significant GO terms observed in mutants were related to mitochondrial genes and metabolism (Supplementary Fig. 4c). Previously, by studying retinal progenitor CKOs, we determined that RONIN regulates a subset of mitochondrial genes impacting function of the electron transport chain (ETC)^23^. However, the E18.5 *Ronin*^*F80L/F80L*^ brains did not show a similar ETC deficiency or changes in mitochondrial content, which suggests this is likely not the cause of the observed phenotypes (Supplementary Fig. 6a, b).

To identify dysregulated genes in the *Ronin*^*F80L/F80L*^ mice directly targeted by RONIN, we performed RONIN ChIP-seq from wild type E16.5 brains. We mapped 1,219 RONIN peaks, which were primarily localized to promoter regions, and highly enriched for the RONIN binding motif (GFY) (Supplementary Fig. 4d–f and Supplementary Dataset 2, 3). It is worth noting that when we overlapped our published P0 retinal ChIP-seq data with our E16.5 brain ChIP-seq data, we identified 858/1,219 as being specific to the brain dataset (Supplementary Fig. 4g, Supplementary Dataset 4–6)^23^. GO analysis confirmed that metabolism of RNA and translation were the most enriched terms in the brain (Supplementary Fig. 4h, Supplementary Dataset 7). We next overlaid these wild type E16.5 brain ChIP-seq data with the *Ronin*^*F80L/F80L*^ E16.5 brain RNA-seq and once again observed structural constituents of the ribosome as the top hit among reduced transcripts (Supplementary Fig. 4i, Supplementary Dataset 8, 9). A subset of these genes was validated by qrtPCR (Supplementary Fig. 5c). In total, our RNA-seq and ChIP-seq data strongly suggest that RONIN directly promotes the expression of a large group of genes encoding ribosomal subunits and this ability is disrupted by the *Ronin F80L* mutation.

### *Ronin*^*F80L/F80L*^ mice exhibit aberrant protein translation

**Fig. 7:**
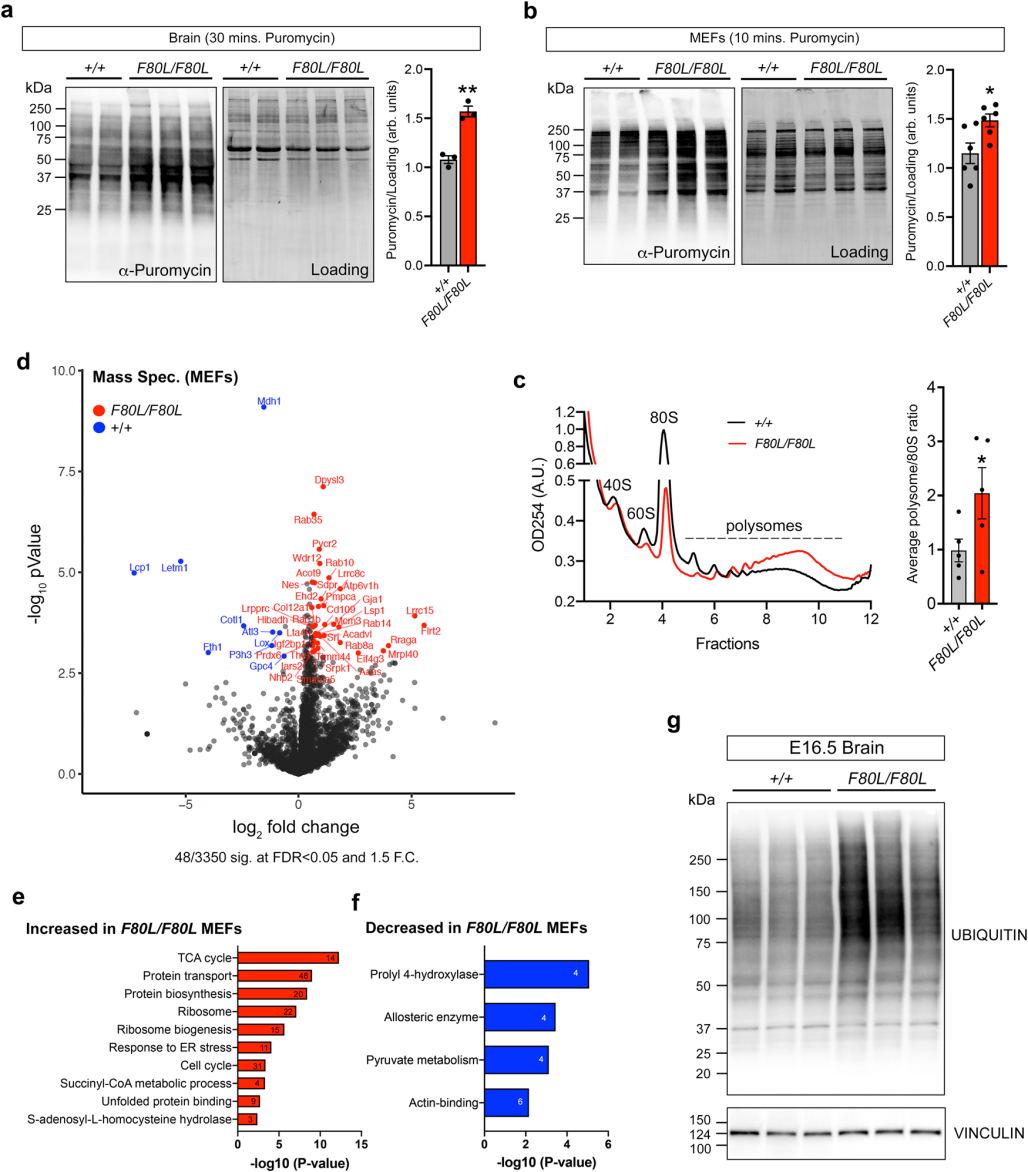
*Ronin*^*F80L/F80L*^ mice have defects in ribosome biogenesis leading to deregulated translation. **a**, **b** Western blot analysis of puromycin incorporation assays. Data are shown as mean ± SEM, *n* = 3 (MEFs) or 6 (brains) wild type, *n* = 3 (MEFs) or 6 (brains) mutants, statistically significant differences between genotypes were determined using the *t* test (two-tailed). **p = 0.002, *p = 0.02. **c** Polysome profiling of wild type and *Ronin*^*F80L/F80L*^ E18.5 brains. Data are shown as mean ± SEM, *n* = 5 wild type, *n* = 5 mutants, statistically significant differences between genotypes were determined using the *t* test (two-tailed). *p = 0.047. **d** Volcano plot of the *Ronin*^*F80L/F80L*^ MEF protein mass spectrometry. **e**, **f** GO analysis of the proteins upregulated and downregulated in the *Ronin*^*F80L/F80L*^ MEF mass spectrometry. **g** Western blot of increased protein ubiquitination in E16.5 *Ronin*^*F80L/F80L*^ brains. Source data are provided as a Source data file.

To determine whether the reduction in transcripts encoding ribosome protein subunits results in a functional deficiency of global translation in the *Ronin*^*F80L/F80L*^ mice, we performed puromycin assays. Mutant and control brain tissue and MEFs were treated with puromycin, which is incorporated into nascent polypeptides and detected by Western blot. Surprisingly, both mutant brain tissue and MEFs showed more intense labeling with anti-puromycin antibodies suggesting a higher-than-normal level of translation (Fig. 7a, b). We next performed polysome profiling of mutant and control E18.5 brains and found that the *Ronin*^*F80L/F80L*^ mutants had a lower 80S ribosome subunit peak and higher polysome peaks also consistent with an increase in translation (Fig. 7c). For a more global assessment of protein abundance, we performed mass spectrometry on lysates from *Ronin*^*F80L/F80L*^ and wild type MEFs and found that 188/3350 proteins (FDR < 0.25 and 2F.C.) were increased in the mutants (Fig. 7d and Supplementary Dataset 10). GO analysis showed the expected upregulation of enzymes within the TCA and methylation cycles that reflect perturbations of MMACHC-dependent cobalamin metabolism (Fig. 7e and Supplementary Dataset 11). On the downregulated side, the GO term “pyruvate metabolism” was present and included Lactate dehydrogenase A (LDHA), which catalyzes the inter-conversion of pyruvate and L-lactate (Fig. 7f and Supplementary Dataset 12). Since pyruvate is a key intermediate for the TCA cycle, the LDHA reduction may be an attempt to reduce pyruvate to L-lactate conversion as a means to divert more pyruvate to the deficient TCA cycle observed in patients with cobalamin disorders. Further supporting the puromycin and polysome data, we also found that factors involved in protein biosynthesis were upregulated. However, we unexpectedly observed overrepresentation of ribosome biogenesis factors and subunit components—some of which are RONIN targets genes (Fig. 7e). Considering the reduced ribosomal subunit gene expression in the *Ronin*^*F80L/F80L*^ mutants, this finding initially seemed counterintuitive, but a recent report of budding yeast ribosome protein mutants has uncovered a similar, unexplained phenomenon (see discussion)^34^. We also performed mass spec. analysis of E18.5 wild type and *Ronin*^*F80L/F80L*^ brains which revealed that this tissue also experiences an increase in proteins involved in ribosome biogenesis and protein synthesis (Supplementary Fig. 7, Supplementary Dataset 13–15). Finally, we performed the SUnSET-based Ribosome Speed of Elongation (SunRiSE) assay on MEFs derived from wild type and *Ronin*^*F80L/F80L*^ mice^50^. We did not uncover any obvious difference between mutant and control cells suggesting that the translation elongation rate is not affected (Supplementary Fig. 8).

### *Ronin F80L* and *Hcfc1 A115V* mutants exhibit phenotypes associated with ribosomopathies

Based on our RNA-seq data showing a reduction in genes encoding ribosome subunits, the apparent increase in ribosome biogenesis and translation in the *Ronin*^*F80L/F80L*^ mutants seemed paradoxical. Nevertheless, it remained possible these processes are still defective and contribute to disease. The first indication that *Ronin F80L* and *Hcfc1* A115V mutants may suffer from pathological changes in translation came from our mass spectrometry data, which revealed an increase in proteins known to function in unfolded protein binding (Fig. 7e, Supplementary Dataset 11). We also found that global protein ubiquitination was increased in the E16.5 *Ronin*^*F80L/F80L*^ brains (Fig. 7g) and plasma amino acid levels were elevated in adult *Hcfc1*^*A115V/Y*^ mice (Supplementary Fig. 9). Together, these data support the idea that this apparent increase in translation may result in aberrant proteins that must be degraded to avoid proteotoxic stress.

**Fig. 8:**
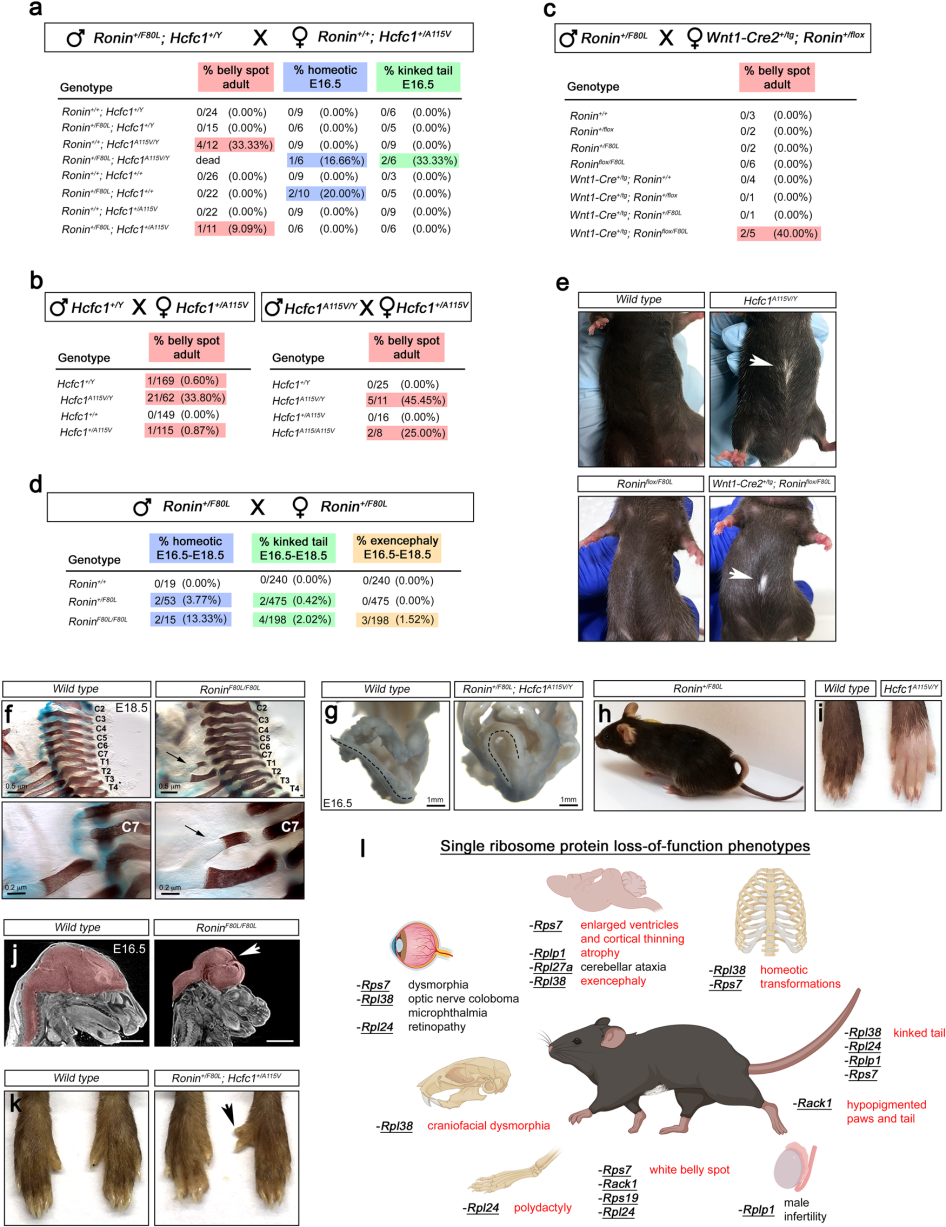
Ribosomopathy phenotypes in *Hcfc1* and Ronin mutants. **a**–**d** Crosses and penetrance of ribosomopathy-associated phenotypes. **e** White belly spotting phenotype. **f** Homeotic transformation of C7 to T1 indicated by an ectopic rib on C7 (arrows). **g**, **h** Kinked tails in *Ronin*^*+/F80L*^*; Hcfc1*^*A115V/Y*^ embryos and a single *Ronin*^*+/F80L*^ mouse. **i** Hypopigmented paws on *Hcfc1*^*A115V/Y*^ adults. **j** Exencephaly in *Ronin*^*F80L/F80L*^ embryos. **k** Polydactyly in a *Ronin*^*+/F80L*^*; Hcfc1*^*+/A115V*^ mouse. **l** Summary of phenotypes reported in single ribosome protein mutants. Phenotypes listed in red were observed in *Ronin* and *Hcfc1* mutants, but never in wild type mice. Panel **l** was created with BioRender.com.

To further investigate the possibility that disrupted ribosome biogenesis and aberrant protein translation contribute to the pathophysiology within our models, we performed crosses between *Ronin* F80L and *Hcfc1* A115V mutant alleles and uncovered a variety of distinct, variably penetrant phenotypes that are typically observed in mouse ribosome protein mutants. In addition to craniofacial dysmorphia (Figs. 4, 5), these defects included white belly spotting, hypopigmented paws and tails, kinked tails, homeotic transformations, exencephaly, and polydactyly (Fig. 8)^25–32^. The most penetrant of these phenotypes was ventral white belly spotting and hypopigmented paws, which were also observed in *Hcfc1 A115V* intercrosses (Fig. 8a, b, e, i). Crosses between *Wnt1-Cre2*^*+/tg*^*; Ronin*^*+/flox*^ and *Ronin*^*+/F80L*^ mice resulted in *Wnt1-Cre2*^*+/tg*^*; Ronin*^*flox/F80L*^ mice that also had white belly spots and hypopigmented paws, supporting a neural crest origin of the phenotype (Fig. 8c, e). Skeletal preparations revealed homeotic transformations across *Ronin F80L* and *Hcfc1 A115V* homo, hetero, and hemizygous genotypes that were present at a low penetrance, but never observed in wild type littermates (Fig. 8a, d, f). These defects included an ectopic rib on cervical vertebrae 7 (2 *Ronin*^*+/F80L*^ and 1 *Ronin*^*F80L/F80L*^) and thoracic vertebrae 11 (1 *Ronin*^*+/F80L*^*; Hcfc1*^*A115V/Y*^), fusion of cervical vertebrae 2 and 3 (1 *Ronin*^*F80L/F80L*^), and a missing lumbar vertebrae 6 (1 *Ronin*^*+/F80L*^). In ribosome protein mutants, similar phenotypes have been shown to be due to anterior homeotic transformations^25,28^. The kinked tail phenotype is typically ascribed to disruptions in somite development, which subsequently impacts axial skeleton formation (Fig. 8a, d, g and h). Exencephaly was a rare phenotype that was only observed in 3/198 *Ronin*^*F80L/F80L*^ embryos (Fig. 8d, j, arrow) and polydactyly was observed in only one *Ronin*^*+/F80L*^*; Hcfc1*^*+/A115V*^ double heterozygous mouse (Fig. 8k, arrow). Taken together, these data suggest that loss of RONIN and HCFC1 activity indeed compromises ribosome activity resulting in a spectrum of specific phenotypes often observed in mice with mutations in genes encoding ribosome subunits (Fig. 8l)^25–32^.

### The *Hcfc1 A115V* and *Rpl24 Bst* alleles genetically interact

**Fig. 9:**
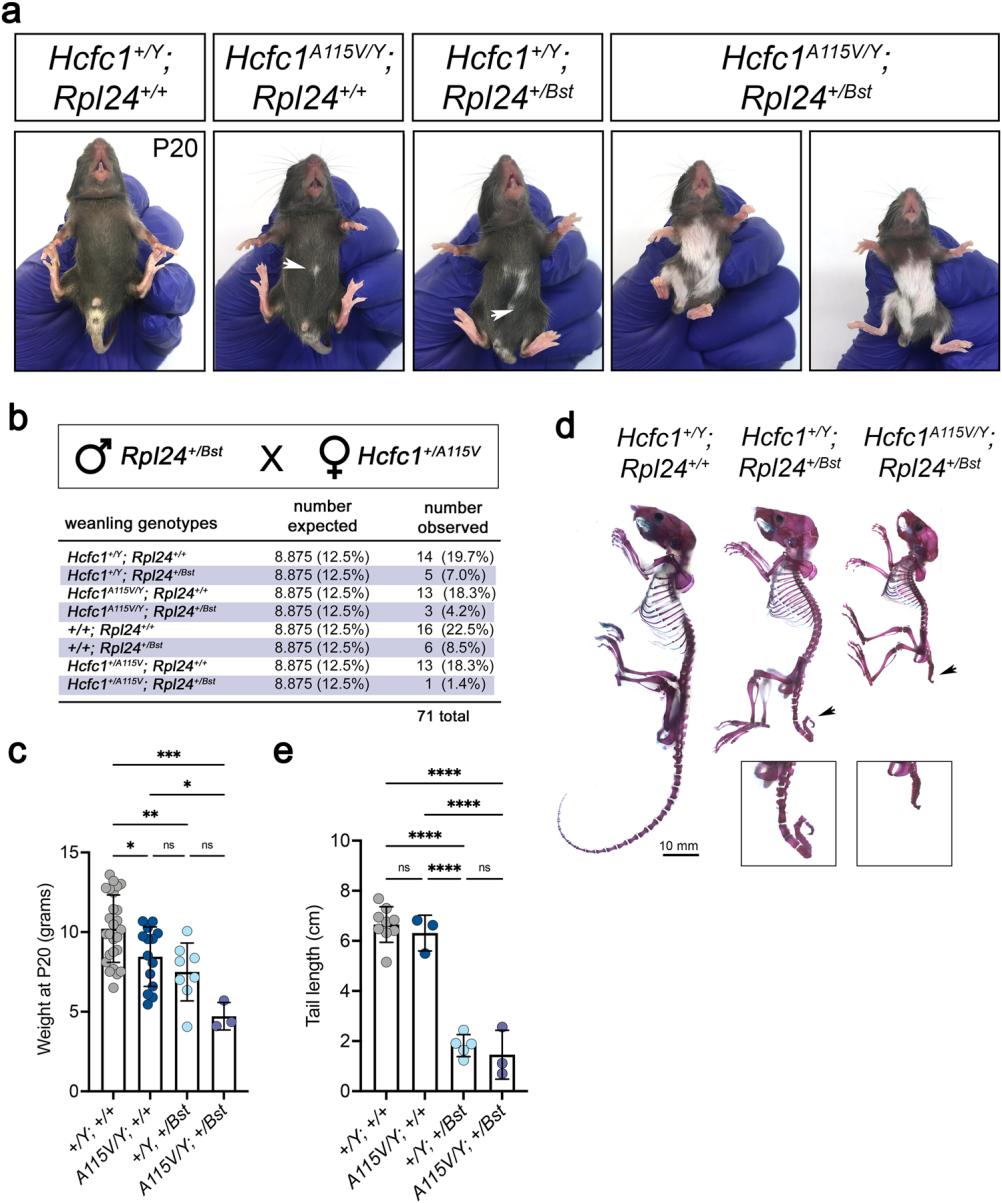
The *Hcfc1 A115V* and *Rpl24 Bst* alleles genetically interact. **a** Expanded belly spot in the *Hcfc1*^*A115V/Y*^*; Rpl24*^*+/Bst*^ mice. **b** Results of genetic crosses between *Rpl24*^*+/Bst*^ and *Hcfc1*^*+/A115V*^ mice. **c** Weight measurements showing a trend of increased runtedness in the *Hcfc1*^*A115V/Y*^*; Rpl24*^*+/Bst*^ mice as compared to *Hcfc1*^*+/A115V*^ and *Rpl24*^*+/Bst*^. **d**, **e** Skeleton preparations and tail length measurements showing that 2/3 *Hcfc1*^*A115V/Y*^*; Rpl24*^*+/Bst*^ mice exhibit a more severe shortening of the tail as compared to the *Rpl24*^*+/Bst*^ mice. All quantified data are shown as mean ± SEM and *n* ≥ 3 biologically independent samples per genotype. Statistically significant differences between genotypes were determined using ANOVA and Tukey’s multiple comparisons test, *p < 0.05, **p < 0.01, ***p < 0.001, ****p < 0.0001. Source data are provided as a Source data file.

As a final assessment of ribosome function in vivo, we crossed the *Hcfc1*^*+/A115V*^ mice to the classical belly spot and tail (Bst) mutant^51^. These mice contain a semidominant homozygous lethal mutation in the ribosome protein gene *Rpl24* that impairs *Rpl24* mRNA splicing and RPL24 protein levels^29^. Heterozygous *Rpl24*^*+/Bst*^ mice are runted, exhibit white belly spotting and a kinked tail, and have deficiencies in ribosome biogenesis and translation^29^. Out of 11 litters, we were able to recover 3 viable *Hcfc1*^*A115V/Y*^; *Rpl24*^*+/Bst*^ mice and these mutants exhibited a dramatic expansion of the white belly spot as compared to the *Hcfc1*^*A115V/Y*^ and *Rpl24*^*+/Bst*^ littermates (Fig. 9a, b). The double mutants also appeared smaller than most of the single mutants (Fig. 9c), and 2/3 had tails that were shorter than the *Rpl24*^*+/Bst*^ mice (Fig. 9d, arrowheads and e). These data underscore our conclusion that, whatever consequence mutations in *Ronin* and *Hcfc1* have on ribosome composition and protein translation, ribosome function is clearly compromised, and results in a ribosomopathy impacting development.

## Discussion

Two *cblC*-like cobalamin deficiency disorders have been recently identified and ascribed to mutations in the genes encoding the transcription factor RONIN (THAP11) and its obligate cofactor HCFC1^20,21^. To better understand the pathophysiology of these diseases, we have generated *Hcfc1*^*A115V/Y*^ and *Ronin*^*F80L/F80L*^ mouse models. These mutant mice exhibited the expected congenital homocystinuria and methylmalonic acidemia, which are the metabolic hallmarks of a cobalamin deficiency syndrome. They also suffered from intrauterine growth restriction, hydrocephalus, anemia, and congenital heart malformations, which are frequently reported in *cblC* patients^9,10^. While the *Ronin*^*F80L/F80L*^ mice die within a few minutes after birth, the *Hcfc1*^*A115V/Y*^ mice are sub-viable with approximately half dying by weaning. The cause of death in both the *Ronin* and *Hcfc1* mutants is currently unclear, but the congenital heart malformations are a possible explanation as conditional knockout of *Ronin* in developing cardiomyocytes resulted in lethality by E13.5^52^. Since this allele deletes the entire *Ronin* coding region, the cardiac defects resulting from the *Ronin F80L* and *Hcfc1 A115V* putative hypomorphic point mutations would be expected to be less severe and possibly result in later onset of lethality. It is important to note that the exact consequence of the *Ronin* and *Hcfc1* point mutations on protein function remains unclear. We have determined that, despite the RONIN F80L protein being unstable, it still binds its transactivator HCFC1. Since the F80L mutation is in the THAP DNA binding domain of RONIN, it is likely that the RONIN F80L/HCFC1 complex cannot efficiently bind DNA. We also found that the HCFC1 A115V protein, which is mutated in a putative Kelch protein-protein interaction domain, still binds RONIN. Since RONIN and HCFC1 exist in a large (>2MDa) protein complex that is not well-defined, it is likely that interaction with unknown, essential HCFC1 binding partners is disrupted leading to inefficient transactivation of RONIN targets^22^. These proteins might also be mutated in patients with similar *cblC*-like disorders in which the causative genes are currently unknown. For example, one such protein is the transcription factor ZNF143, which was shown to associate with RONIN and HCFC1 at promoter sites. A single patient with an inborn error of cobalamin metabolism was discovered to have reduced *MMACHC* transcription and mutations in *ZNF143*^53–55^. However, we have been unable to detect co-immunoprecipitation between RONIN, HCFC1, and ZNF143 from embryonic mouse brain and retinae suggesting possible tissue- and/or species-specific differences (Supplementary Fig. 10).

Consistent with a recent report of *hcfc1b* morpholino knockdowns, our mouse models exhibited highly penetrant craniofacial dysmorphia^21^. The similarities between the craniofacial anomalies of our *Hcfc1*^*A115V/Y*^ mice and recently identified brothers, also with point mutations in the *HCFC1* kelch domain, was particularly striking^43^. However, our finding that the craniofacial phenotypes observed in our *Ronin* and *Hcfc1* mutants were not due to reduction of *Mmachc* in neural crest cells, which give rise to the majority of the craniofacial skeleton, was unexpected. These data suggested that this phenotype is due to target genes other than *Mmachc*. Though, zebrafish studies suggested that global introduction of human *MMACHC* mRNA is capable of rescuing craniofacial defects observed in *hcfc1b* morpholino knockdowns^56^. We cannot currently explain these species-specific differences, but a recent report of *mmachc* frameshift mutant zebrafish does not describe any craniofacial malformations^57^.

Another unexpected finding was that the embryonic *Ronin*^*F80L/F80L*^ CNS did not exhibit any obvious signs of defective neurogenesis, but that astrocytes appeared reduced. Therefore, it is possible that RONIN may be a regulator of astrogliogenesis, and patients with mutations in either *RONIN* or *HCFC1* suffer from a loss of CNS astrocytes. It is currently unclear to what extent reduced MMACHC activity contributes to the CNS pathology and whether *cblC* patients have a similar defect. Also of note, both morpholino knockdown of *ronin* or overexpression of human *RONIN* in zebrafish led to a reduction in embryonic CNS neuron differentiation, which is not consistent with our findings in the *Ronin*^*F80L/F80L*^ mouse^21^. Even more curious, when mRNA encoding the human RONIN F80L protein was introduced into wild type zebrafish, this increased the population of HuC/D+ neurons, which is the opposite of the *ronin* morpholino phenotype^21^. These seemingly contradictory results remain unclarified. Evidently, mutations in *Ronin*, *Hcfc1*, and *Mmachc* result in complex, pleiotropic phenotypes. If we are to define the precise requirements of these genes in a tissue-specific context during development, future mouse conditional knockout studies are warranted.

Through our RNA-seq and ChIP-seq studies, we have identified RONIN and HCFC1 as direct transcriptional regulators of genes involved in ribosome biogenesis including a cohort encoding large (60 S) and small (40 S) ribosome subunit components. Currently, the most well-studied driver of ribosome biogenesis is the MYC family of transcription factors, which have been described as “master regulators” required for the production of both ribosomal protein and RNA^58–63^. Interestingly, HCFC1 has been previously shown to interact with C-MYC in immortalized cell lines and recently shown to function as a transcriptional cofactor^64,65^. However, we were unable to detect an endogenous interaction between RONIN and C-MYC proteins in the E16.5 mouse brain (Supplementary Fig. 10). Other studies, also in immortalized cells, have suggested that RONIN is a direct transcriptional repressor of *Myc*^66,67^. It is worth noting that neither *Myc* (C-MYC)*, Mycn* (N-MYC), or *Mycl1* (L-MYC) contained RONIN ChIP-seq peaks in the E16.5 mouse brain. Furthermore, *Myc* expression was unchanged in our *Ronin*^*F80L/F80L*^ E16.5 brain RNA-seq dataset, but *Mycn* and *Mycl1* were modestly increased. Therefore, the connection between RONIN, HCFC1, and MYC during ribosome biogenesis remains obscure. It is still possible that a RONIN/HCFC1/MYC complex jointly regulates ribosome biogenesis in vivo. It is also possible that a RONIN/HCFC1 complex functions independently of, or in a cross-regulatory fashion with a MYC/HCFC1 complex.

Our finding that RONIN directly promotes the transcription of genes encoding ribosomal subunit components, and that a large cohort of these genes are transcriptionally reduced in the *Ronin*^*F80L/F80L*^ embryonic brain, raised the possibility that the *HCFC1* and *RONIN* deficiency syndromes are in part ribosomopathies. Ribosomopathies are typically inherited or sporadic disorders due to mutations in genes encoding ribosome biogenesis factors, ribosome protein subunits, and other components of the mature ribosome^68,69^. In recent years it has become known that, despite the ubiquitous requirement for ribosomes in all cells, mutations in different genes essential for ribosome function result in a wide array of tissue specific developmental phenotypes^70^. For example, mutations in *Rplp1* result in brain atrophy, male infertility, and kinked tails whereas the *Rpl38* mutant mice exhibit craniofacial dysmorphia, microphthalmia, exencephaly, and homeotic transformations of the axial skeleton^25,26,70^. If mutations in *Ronin* or *Hcfc1* result in a ribosomopathy, we would expect to observe some of the same phenotypes described for ribosome subunit mutant mice. Indeed, for both *Ronin* and *Hcfc1* mutants, we cataloged a variety of well-known ribosomopathy-associated phenotypes that included white belly spotting, hypopigmented paws and tails, craniofacial dysmorphia, homeotic transformations, polydactyly, and kinked tails^70^. The fact that so many of these phenotypes were present and at variable penetrance may reflect our finding that *Ronin*^*F80L/F80L*^ embryonic brains experience a relatively modest, but broad reduction in genes encoding ribosome subunit components. In other words, the *Ronin*^*F80L/F80L*^ mutants may experience a subtle but global dampening of ribosome subunit protein content leading to dysfunction. It is also worth considering the idea that the cortical thinning and anemia, that we initially ascribed to loss of *Mmachc* expression, might in fact be due to ribosome dysfunction as these phenotypes are also typically observed in ribosomopathies^70^. So far, the only documented phenotype of either the *Ronin*^*F80L/F80L*^ or *Hcfc1*^*A115V/Y*^ mice not typically seen in ribosomopathies, but reported for *cblC* patients, is the ventricular hypertrabeculation and thinning of the myocardium^71^. Interestingly, we have recently characterized similar cardiac defects in *Mmachc* mutant mice^49^. Our findings raise the possibility that *HCFC1* and *RONIN* deficiency syndromes may be primarily ribosomopathies with the cobalamin deficiency playing a less prominent role. If this is indeed the case, it might factor into how these patients are clinically managed relative to *cblC* patients as cobalamin supplementation may have even less efficacy than for *cblC*.

By establishing disorders with mutations in *RONIN* and *HCFC1* as ribosomopathies, it is important to discuss the two prevailing, non-mutually exclusive models that attempt to explain the resulting tissue-specific phenotypes. The longest-standing model is the ribosome concentration hypothesis in which reduced ribosome protein expression leads to defective ribosome assembly and reduced ribosome levels^69,72^. As a consequence, while global translation is usually modestly reduced, specific mRNAs have a greater sensitivity to ribosome concentration versus others and are not translated at sufficient levels. This mechanism was shown to be the case for GATA1 expression in the ribosomopathy Diamond-Blackfan anemia^73^. A second model to explain differential mRNA translation in ribosomopathies is the specialized ribosome hypothesis^69,74^. This model argues that when a particular ribosome subunit is reduced, this changes the overall complement of the ribosome proteins (or modifications to protein or rRNA) within a ribosome. As a result, the ribosome adopts an altered structure and may lose affinity to specific mRNAs while gaining affinity to others leading to shift in the population of mRNA translated within the cell.

A third possible explanation, related to the two mentioned above, is that changes in ribosome quantity or composition might elicit a maladaptive, compensatory transcriptional response that results in the expression of different mRNAs within the cell thereby contributing to tissue-specific phenotypes. Recently, this idea was investigated in a systematic study of different budding yeast ribosome protein deletion mutants and their respective gene expression changes^34^. Yeast, which incidentally do not have cobalamin, is an excellent model system in which to perform this investigation because they lack a p53-like checkpoint that typically causes secondary gene expression changes in response to ribosome protein deficiency. Therefore, any observed transcriptional changes may be more enriched for those directly related to the ribosome deficiency itself. While they indeed uncovered robust general gene expression signatures due to loss of ribosome proteins, they also made the surprising discovery that the most dramatic transcriptional changes within each mutant differed on whether a large (60S) or small (40S) subunit component was missing. In *rpl* 60S mutants, they observed specific upregulation of proteasome-mediated degradation factors. This increase was interpreted as possibly being due to the fact that *rpl* mutants degrade both super-stoichiometric 60S and 40S subunits and therefore require additional proteasome activity. Consistent with this idea, the yeast ubiquitin-proteasome system was previously shown to degrade ribosomes that are produced in excess^75^. Another possible explanation is that the 60S mutants are more likely than 40S mutants to result in the production of poor-quality nascent proteins, which increases the cellular demand for degradation. In the case of the *rps* 40S mutants, they found that genes involved in ribosome biogenesis were upregulated, and despite being growth-defective, the cells accumulated mature 60S subunits. There is currently no explanation as to why the *rps* mutants keep 60S components while degrading 40S components, and yet continue to synthesize more of both. Also, the biological consequence of these phenotypic differences between *rpl* and *rps* mutants is unknown. However, recall that RONIN directly promotes the expression of genes encoding both 60S and 40S components and that our *Ronin*^*F80L/F80L*^ mass spectrometry data point to an increase in both unfolded protein binding and ribosome biogenesis. Furthermore, our puromycin assays and polysome profiling consistent with an increase in translation, yet we also observed an increase in global protein ubiquitination and free amino acids. In total these data suggest that the embryonic *Ronin*^*F80L/F80L*^ brain exhibits features of both 60S and 40S loss that were described in yeast mutants.

To conclude, similar to its role in the maintenance of ESC growth, we have established RONIN and HCFC1 as essential regulators of ribosome biogenesis during embryonic development^76^. While the exact molecular underpinnings are likely complex and remain to be resolved, based on the tissue-specific phenotypes of our *Ronin* and *Hcfc1* mutant mice, we also make a strong case that diseases caused by mutations in these genes are ribosomopathies. Despite translation appearing to be grossly increased in our *Ronin*^*F80L/F80L*^ mutants, it is possible that overall ribosome concentration per cell, which is very difficult to measure, is still reduced and impacts the translation of specific mRNAs sensitive to the reduction. Alternatively, it may be that subtle reduction in the availability of a subset of ribosome subunit components that are RONIN targets leads to alterations in overall ribosome stoichiometry throughout the embryo. This shift in ribosome components may re-specialize the mutant ribosome leading to a loss of translation of some mRNAs and inappropriate translation of others for which the ribosome has acquired affinity. Whatever the case, our findings are consistent with both the concentration and specialization models of ribosomopathies. Future studies will shed further light on the precise consequence on protein translation and the pathophysiology of these transcription factor diseases.

## Methods

### Mice strains and genotyping

Our study protocol was approved by the Institutional Animal Care and Use Committee (IACUC) of Baylor College of Medicine. Mice were housed in static micro-isolator cages within an AAALAC-accredited facility in compliance with the *Guide for the Care and Use of Laboratory Animals*, 9th edition. They were maintained on a 12:12-hour light:dark cycle at a temperature of 70–73 °F and a relative humidity of 30–70%. Mice were provided food (LabDiet, #5V5R) ad libitum and water bottles with chlorine dioxide treated water. Cages were changed weekly, and all mice were provided an Enviropak for enrichment at each cage change. Mice were weaned at 3 weeks of age and euthanized at the desired age for experiments. Since there were no age- or sex-dependent differences in our observations, male and female data were combined for presentation herein with the exception of *Hcfc1* A115V hemizygous males compared to wild type males. Mice were maintained on C57BL/6J genetic background.

To genotype the *Ronin*^*+/F80L*^ and *Ronin*^*F80L/F80L*^ mice, genomic tail DNA was PCR amplified with the following primers and cycle conditions: F80L_F: 5′-CCTCTGGCTCAAGAACGTGT-3′ and F80L-R: 5′-ATGGGCTTCAACATCATCTCC-3′, 95 °C/5 min; 95 °C/30 s; 60 °C/30 s; 72 °C/45 s; cycles 2–4 34×, 72 °C/10 min, 4 °C hold. The 435 bp product was then analyzed with the Surveyor Mutation Detection Kit (Integrated DNA Technologies, IDT, #706020). The presence of 296 bp and 139 bp bands indicated the *F80L* allele. The *Ronin* floxed allele was was gifted to our lab by Dr. Thomas Zwaka and genotyped using the following primers and conditions to generate either a 160 bp (WT) or 200 bp (floxed) product: Rnfx_F: 5′-TACCCAGAGCGCTTGCGCTCACCAG-3′ Rnfx_R: 5′-TCCAGATGAAGCTCGTCCTAAGCGA-3′ 95 °C/5 min; 95 °C/30 s; 58 °C/30 s; 72 °C/30 s; cycles 2–4 34×, 72 °C/10 min, 4 °C hold^22^. To genotype the *Hcfc1*^*A115V/Y*^ mice, the following primers and cycle conditions were used: A115V_F 5′-TCGCTCCTTGAACAGTGTCA-3′ and A115V_R 5′-AGCTATGTCCAAGCCGAGGA-3′, 95 °C/5 min; 95 °C/30 s; 60 °C/30 s; 72 °C/45 s; cycles 2–4 34×, 72 °C/10 min, 4 °C hold. The 248 bp product was then digested with BccI restriction enzyme (37 °C for 1 h). The A115V mutant allele contains a silent mutation generating a BccI site thereby giving rise to 80 bp and 168 bp products. The *Mmachc*^*flox/flox*^ and *Mmachc-OE*^*+/tg*^ mouse lines were recently generated and functionally validated by our lab. Details regarding the generation and genotyping of these mice are reported in Chern, et al. ^49^. To genotype the *Mmachc*^*flox/flox*^ and *Mmachc-OE*^*+/tg*^, the following primers and cycle conditions were used: MLox_F1 5′-TCTCCTTGCTGCCATCTTGG-3′ and MLox_R1 5′-TAGTAGGAGAGAGGTTGGGC-3′ (to detect a 174 bp WT band and 212 bp floxed band), and GFP_F 5′-AAGTTCATCTGCACCACCG-3′ and GFP_R 5′-TCCTTGAAGAAGATGGTGCG-3′, 95 °C/5 min; 95 °C/30 s; 57 °C/30 s; 72 °C/45 s; cycles 2–4 34×, 72 °C/10 min, 4 °C hold. The *Wnt1-Cre2*^*+/tg*^ (JAX 022137); *Prrx1-Cre*^*+/tg*^ (JAX 005584); *Krox20-Cre*^*+/tg*^ (JAX 025744); and *Phox2b-Cre*^*+/tg*^ (JAX 016223) lines were obtained from the Jackson Laboratory and genotyped with the following primers and cycle conditions resulting in a 219 bp product: Cre_F: 5′-GGACATGTTCAGGGATCGCCAGGC-3′ and Cre_R: 5′-CGACGATGAAGCATGTTTAGCTG-3′, 95 °C/5 min; 95 °C/30 s; 57 °C/30 s; 72 °C/45 s; cycles 2–4 34×, 72 °C/10 min, 4 °C hold^35,47,48,77^. The *Rpl24*^*+/Bst*^ line was genotyped with the following primers and cycle conditions resulting in a 562 bp product for wild-type allele and a 590 bp product for the *Bst* allele: Rpl24-WT_F: 5′-CTCTTTGCAGCGCACATACTAAC-3′, Rpl24-WT_R: 5′-GGAAAACCTGCAGTTAACAAATTC-3′, Rpl24-Bst_F: 5′-TTTGCAGCGCACATACGAG-3′, Rpl24-Bst_R: 5′-GCTGACTCACATTTTGCATTAAGA-3′, 95 °C/5 min; 95 °C/30 s; 56 °C/30 s; 72 °C/45 s; cycles 2–4 34×, 72 °C/10 min, 4 °C hold^29^. For all PCR genotyping reactions, the following primers and cycle conditions were used as an internal control and gave rise to a 590 bp product: Rap_F: 5′ -AGGACTGGGTGGCTTCCAACTCCCAGACAC-3′ and Rap_R: 5′-AGCTTCTCATTGCTGCGCGCCAGGTTCAGG-3′, 95 °C/5 min; 95 °C/30 s; 57 °C/30 s; 72 °C/45 s; cycles 2–4 34×, 72 °C/10 min, 4 °C hold. All primers were purchased from Sigma-Aldrich.

### Generation of mouse models

The *Ronin*^*F80L/F80L*^ and *Hcfc1*^*A115V/Y*^ mouse lines were generated using CRISPR/Cas9 genome editing. Single guide RNA (sgRNA), single-stranded oligonucleotides (ssOligo, DNA donor), and Cas9 mRNA were purchased from IDT, Inc. and microinjected into single-cell C57BL/6J mouse zygotes by the Baylor College of Medicine Mouse Embryonic Stem Cell Core. Mice with putative correct targeting were first identified with the Surveyor Mutation Detection Kit (IDT). Mice showing a DNA mismatch with Surveyor were then validated by sequencing a 1 kb region around the intended nucleotide substitution sites. A subset of founders showing only the specific, targeted point mutations were bred to C57BL/6J mice and F1 offspring sequenced to confirm germline transmission of the point mutations. Prior to phenotyping studies, the F1 carriers were backcrossed to C57BL/6J for a minimum of 4 generations. We also established congenic lines that were backcrossed to C57BL/6J for 10 generations. Further details on the CRISPR/Cas9 genome editing strategy are available upon request.

### Mouse embryonic fibroblast (MEF) collection

E13.5-E15.5 bodies were collected from control, *Ronin*^*F80L/F80L*^, and *Hcfc1*^*A115V/Y*^ embryos and the livers and hearts were removed. The remaining tissue was transferred into a clean petri dish, and 300 µL of 0.05% trypsin-EDTA was added (Gibco, #25200056). The tissue was then minced using a clean razor blade for 5 to 7 min until single cells were released. The sample was then pipetted into a 15-mL conical tube containing 5 mL DMEM growth medium (Gibco, #11995065) with 10% fetal bovine serum (FBS) (Gibco, #26140079) and 1% penicillin/streptomycin (Pen/Strep) (Gibco, #15140122). After allowing the tissue to settle for 2 min, the supernatant with single cells was transferred to another clean 15-mL conical tube and centrifuged at room temperature (150 × *g*, 5 min). The media was removed, and the cell pellet was resuspended with 10 mL fresh DMEM. Cell suspension was then seeded into a clean 10-cm cell culture dish and incubated at 37 °C with 5% CO_2_ overnight before changing media and cell expansion.

### [^57^Co] cobalamin distribution, and [^14^C] propionate and [^14^C] methyltetrahydrofolate (methylTHF) incorporation

MEFs from wild type, *Ronin*^*F80L/F80L*^, and *Hcfc1*^*A115V/Y*^ embryos were cultured in modified Eagle minimum essential medium plus non-essential amino acids (Wisent, St Bruno QC) supplemented with 15% (v/v) fetal bovine serum (Wisent). Cells were incubated for 96 hrs. in medium supplemented with 25 pg/mL [^57^Co]cyanocobalamin (MP Biomedicals, Solon OH) bound to murine transcobalamin in mouse serum. At the end of the incubation, cells were harvested by trypsinization and intracellular cobalamin extracted in hot ethanol. Cobalamin coenzyme forms were separated on a LUNA 100 Å C8 high performance liquid chromatography column (Phenomenex, Torrance CA) on a gradient of 13–35% acetonitrile in 0.05 M phosphoric acid buffer pH3^78^. Fractions of column eluate were collected and analyzed by gamma counting. Cobalamin derivatives were identified by coelution with hydroxocobalamin, cyanocobalamin, adenosylcobalamin, and methylcobalamin.

Functional activity of methylmalonyl-CoA mutase in intact mouse embryonic fibroblasts was assessed by measurement of incorporation of label from propionate into trichloroacetic acid-precipitable material. Fibroblast cultures were set up at a density of 400,000 cells per 35-mm tissue culture dish and incubated for 18 hrs. in Puck’s F medium supplemented with 15% fetal bovine serum and 100 µmol/L [1-^14^C]propionate (Pharmaron special synthesis, Cardiff UK; diluted with cold propionate to give a final specific activity of 10 µCI/µmol). The assay was performed in the presence and absence of 3.75 µM hydroxocobalamin. At the end of the incubation, macromolecules were precipitated with 5% trichloroacetic acid. The precipitated material was dissolved in 0.2 N sodium hydroxide and radioactivity was determined by liquid scintillation counting^79^.

Functional activity of methionine synthase was determined by measurement of incorporation of label from 5-methyltetrahydrofolate into trichloroacetic acid-precipitable material. MEF cultures were set up as for propionate incorporation assay and incubated for 18 h in methionine-free minimum essential medium (Wisent) supplemented with 200 µM homocysteine thiolactone, 50 µg/mL sodium ascorbate and 10% dialyzed fetal bovine serum, in which folic acid had been replaced by 5-[^14^C]methyltetrahydrofolate (Pharmaron special synthesis, Cardiff UK; 0.5 µCi/mL, 60 mCi/mmol). The assay was performed in the presence and absence of 3.75 µM hydroxocobalamin. At the end of the incubation, macromolecules were precipitated with 5% trichloroacetic acid. The precipitated material was dissolved in 0.2 N sodium hydroxide and radioactivity was determined by liquid scintillation counting^79^.

### Luciferase reporter assay

To examine whether RONIN directly binds to the putative RONIN binding motif within the *Mmachc* promoter region, we used the Dual-Glo Luciferase Assay System (Promega, #E2920). HeLa cells were seeded into a 96-well plate with relative equal number per well and incubated at 37 °C and 5% CO_2_ overnight until 60–70% confluent. Using the Lipofectamine-3000 system (Invitrogen, #L3000001) in 5 µL Opti-MEM (Invitrogen, #31985062), the cells were then transfected with 15 ng of experimental reporter plasmids (*Ronin-motif-luc* or *Empty-luc*), 31, 62, or 94 ng of *CMV-Ronin* or *CMV-Empty* control plasmid, and 3.3 ng *Renilla* plasmid (control reporter) After overnight incubation, the media was removed from wells and the remaining cells were equilibrated to room temperature. 75 µL Dual-Glo Reagent was then added to each well, incubated for 10 min at room temperature, and the luciferase luminescence was measured with a plate reader. Then, 75 µL Dual-Glo Stop & Glo Reagent was added and mixed to each well, incubated for 10 min, and the control Renilla luminescence was measured with the same plate reader. To analyze the data, the luciferase luminescence was divided by the Renilla luminescence to generate the relative luminescence ratio and then normalized to the ratio from the control well. The Student’s *t*-test was used to determine differences between control and *Ronin* overexpressing groups.

### Measurement of mitochondrial DNA (mtDNA)

To measure wild type, *Ronin*^*+/F80L*^, and *Ronin*^*F80L/F80L*^ mtDNA content, we used the NovaQUANT^TM^ Mouse Mitochondrial to Nuclear Ratio Kit (Millipore, #72621). DNA from E16.5 brains were extracted using the Gentra Puregene Tissue Kit (Qiagen, #158667). The eluted DNA was diluted to 0.2 ng/µL and used as a template for qPCR using a StepOnePlus Real-Time PCR System (Life Technologies) and following the NovaQUANT^TM^ manufacturer’s instructions and thermal cycling conditions appropriate for Fast Block and the RT^2^ Fast SYBR Green Mastermix (SABiosciences, #PA-042). Four independent assays were performed on three independent DNA samples per genotype. To analyze the data, we used the Relative Copy Number Method and the data were represented as the average number of copies of mtDNA. The Student’s *t*-test was used to determine differences between wild type and *Ronin*^*+/F80L*^ or *Ronin*^*F80L/F80L*^.

### Craniofacial morphometric analysis

Landmark-free morphometrics analysis was performed as previously described^45^. Briefly, micro-CT scans were converted into three-dimensional meshes and then aligned using a Procrustes superimposition removing scale differences. The software Deformetrica was then used to “atlas” the entire population to quantify the shape of each individual and visualized graphically through principal component analysis (PCA) to find the principal modes of shape variation^80^. Morphs were generated by projecting the average mesh into the PCA shape space and deforming towards the means. Centroid sizes were measured and normalized as the square root of the sum of the squared means from each node of the mesh to the centroid divided by the number of nodes^81^. Heat maps were generated in paraview (ISBN-13: 978-0123875822) using the average morph and color coding whether the mesh polygons decrease or increase in area. Stretch heatmaps were generated by calculating local mesh node density changes under the Deformetrica-generated displacements using previously written code^45^. Nasal angles were measured by the deviation from the midline from the superior views of each skull using ImageJ. Linear measurements of the craniofacial bones were performed as previously described using ImageJ^44^. One-way ANOVA with post-hoc Tukey’s multiple comparisons test was used to determine differences between more than 2 groups. Adjusted *P* values of <0.05 were considered significantly different.

### Amino acid and metabolite measurements

Blood from wild type, *Hcfc1*^*A115V/Y*^, and *Hcfc1*^*A115V/Y*^*; Mmachc-OE*^*+/tg*^ adult mice was collected retro-orbitally using heparinized capillary tubes (Thermo Fisher, #22-260950). The samples were then centrifuged at 4 °C (9600 × *g*, 15 min) and the plasma was transferred to a new tube. The plasma was stored at −80 °C until sent to Biochemical Genetics Laboratory at Baylor Genetics for measurements of amino acid, Hcy, and MMA levels^82–84^.

For amino acid analysis, 30–100 µl of sample was mixed with sample dilute buffer Seraprep (Pickering) and an internal standard (S-2-aminoethyl-1-cysteine). The mixture was then centrifuged for deproteinization. Filtered supernatant was injected into a Hitachi L-8900 Amino Acid Analyzer (Hitachi, Japan) where individual amino acids were separated by ion exchange liquid chromatography. After binding to ninhydrin, the color end products were measured with visible spectrophotometry. The chromatogram was integrated and quantitated with EZchrome software by comparing signal intensities with known standard solutions.

Total plasma homocysteine and methionine were measured by HPLC-MS/MS (Waters, Quattro Micro, UK). 50–100 µl of sample was mixed with isotopic internal standards (D4-homocysteine and D4-methionine). To free protein bound to homocysteine, 20 µl of 500 mM dithiothreitol was added to samples and incubated for 30 min at 65 °C. Next, 280 µl of cold TPH loading buffer is added before loading to mass spec. Calibration curves were used to quantitate analytes with Waters QuantiLynx software.

Methylmalonic acid was measured by HPLC-MS/MS (Waters, Quattro Micro, UK). 500 µl of sample was mixed with D3-methylmalonic acid and 90 µl of phosphoric acid. Next, methylmalonic acid was extracted in the presence of tert-butyl methyl ether (MTBE). After centrifugation, supernatant was collected in a new tube and dried. Butanoic acid derivatization was performed. The final dried butylated product was dissolved in 85% methanol. Details of sample preparation and LCMS settings can be found in reference below. Calibration curves were used to quantitate analytes with Waters QuantiLynx software. One-way ANOVA with post-hoc Tukey’s multiple comparisons test was used to determine differences between more than 2 groups. Adjusted *P* values of <0.05 were considered significantly different.

Quality assurance and quality control (QA/QC) procedures: All tests are performed in a clinical laboratory that meets all CLIA/CAP QA/QC requirements. In brief, commercial low, medium, and high controls are run with samples in each batch. Acceptable control ranges are set by running samples at least 10 times. Result statistics was performed, and the data coefficient of variation has to be within 15% for acceptance. A 2 SD range was set as acceptable control values. Control values in each experimental batch needs to meet pre-determined acceptable ranges. Control values are reviewed monthly to make sure no apparent abnormal trends or shifts are observed. Periodic calibrations are performed using College of American Pathology (CAP) proficiency surveys. Internal standards are added in each sample to normalize variation caused in preparation or analysis. Test performance, such as precision, accuracy, linearity of analytical ranges, and reproducibility, are checked twice a year.

### Blood cell counts

Using a heparinized capillary tube, blood was collected from decapitated E18.5 wild type, *Ronin*^*+/F80L*^, *Ronin*^*F80L/F80L*^, and *Hcfc1*^*A115V/Y*^ mice and transferred into a 1.5 mL tube containing 1.5 μl of 0.5 M EDTA. The blood was then run through an automated hematology analyzer (Scil Vet abc animal blood counter) to obtain 13 hematologic parameters including red blood cell count, white blood cells, hematocrit, hemoglobin, platelets, and mean corpuscular volume (MCV).

### Tissue collection, histology, and image acquisition

E16.5 and E18.5 brains, hearts, whole heads, and whole bodies were dissected in cold PBS and fixed in cold 4% paraformaldehyde (PFA) at 4 °C for 4 h. After 3 × 10 min washes with PBS, they were incubated in a 15% sucrose solution (made in PBS) overnight at 4 °C, and then in a 30% sucrose solution for an additional night at 4 °C. The tissue was then submerged in OCT compound (Sakura, #4583), frozen on dry ice, cryosectioned at 20 µm, and mounted on Superfrost Plus slides (VWR). Some slides were processed for H&E staining and coverslips were mounted with DPX New (Millipore, #1005790507). Immunofluorescence was performed as follows: slides with cryosections were thawed at room temperature for 10 min and then post-fixed with 4% PFA for 10 min at room temperature. Slides were then blocked with 5% normal goat serum, 3% bovine serum albumin (BSA) and 0.3% Triton-X for 1 h. at room temperature followed by incubation of primary antibodies overnight at 4 °C. The following day the primary antibodies were washed off with 3 × 10 min washes with PBS, followed by incubation of secondary antibodies and DAPI at room temperature for 1 hr. The following primary antibodies were used: GFAP (1:500, DAKO, #Z0334), AQP4 (1:500, Millipore, #AB2218), HuC/D (1:100, Invitrogen, #A-21271). The following secondary antibodies were used: goat, anti-rabbit AlexaFluor 488 and goat, anti-rabbit AlexaFluor 555. Coverslips were then mounted with Fluoromount-G (Southern Biotech, #0100-01). Images of the stained cryosections were taken using a Zeiss LSM 780 Confocal Microscope.

### Micro-computed tomography (micro-CT) sample preparation and scanning

Embryos were dissected in ice-cold PBS and fixed in 4% PFA overnight at 4 °C. After fixation, embryos were transferred to new tubes and immersed in fresh stabilization buffer [4% PFA, 4% acrylamide (Bio-Rad, #161-0140), 0.05% bis-arcylamide (Bio-Rad, #161-0142), 0.25% VA044 initiator (Wako Chemical, #017-19362), 0.05% Saponin (Sigma, #84510) in PBS] for 3 days at 4 °C. Then, sample tubes were capped off and purged with nitrogen at 10 psi for 3 min. Tubes with samples were recapped and incubated for 3 h. at 37 °C for the hydrogel to polymerize. Samples were removed from the hydrogel and stored in PBS with 0.1% sodium azide at 4 °C until ready for imaging. Before micro-CT scanning, embryos were immersed in 0.1 N iodine (Thermo Fisher, #SI86-1) and rocked overnight at room temperature. Micro-CT images were acquired using a Bruker SkyScan 1272 Scanner at a 11μm resolution. Adult skulls from wild type, *Hcfc1*^*A115V/Y*^, and *Hcfc1*^*A115V/Y*^*; Mmachc-OE*^*+/tg*^ mice were similarly prepared, but without iodine immersion.

### Bone and cartilage staining

Embryos were dissected and the skin and viscera were removed followed by dehydration in 95% ethanol overnight at 4 °C. The next day the embryos were incubated with stain base solution (70% ethanol, 5% acetic acid) for 30 min, and then stained in 70% ethanol, 5% acetic acid, 0.02% alcian blue, 0.05% alizarin red for 24 h. rocking at room temperature. Following staining, embryos were rinsed and incubated in stain base solution for 30 min, rinsed in water, incubated in 2.0% potassium hydroxide (KOH) on a rocker at room temperature (4–7 h. based on embryonic stage), and cleared in a 0.25% KOH-glycerol series (20% glycerol/0.25% KOH for 1 h, 33% glycerol/0.25% KOH for 1 h, 50% glycerol/0.25% KOH overnight). To preserve the staining embryos were post-fixed with 2% PFA/50% glycerol overnight and stored in 50% glycerol. Staining for adults were similarly performed with longer incubation for each process. Adults were dissected and the skin, organs, and fat tissue were removed followed by dehydration in 95% ethanol for 7 days and dissolving remaining fat in acetone for 7 days rocking at room temperature. Samples were then stained in staining solution for 7 days rocking at room temperature. Following staining, samples were rinsed in water and incubated in 2.0% KOH for 5–7 days and cleared in 0.25% KOH-glycerol series (overnight for each glycerol gradient) rocking at room temperature. The skeletons were then post-fixed with 2% PFA/50% glycerol for 2 days at room temperature and stored in 50% glycerol. Images of the stained skeletons were taken using a Zeiss Axiozoom.

### Western blot analysis

Brains or MEFs from wild type, *Ronin*^*+/F80L*^, *Ronin*^*F80L/F80L*^, and *Hcfc1*^*A115V/Y*^ embryos were homogenized in lysis buffer (20 mM Tris-HCl [pH 7.5], 150 mM NaCl, 1 mM EDTA, 1 mM EGTA, 1% Triton-X, 2.5 mM sodium pyrophosphate, 1 mM β-glycerophosphate, 1 mM Na_3_VO_4_)supplemented with Halt^TM^ Protease Inhibitor Cocktail (ThermoFisher, #78430) and Halt™ Phosphatase Inhibitor Cocktail (ThermoFisher, #78420). Clarified protein lysate was quantified by the Pierce™ BCA Protein Assay (Thermo Fisher, #23225) and 30 µg (90–150 µg if probing for MMACHC) loaded onto a 4–15% Criterion^TM^ TGX precast midi protein gel (BioRad, #567-1084) for electrophoresis and subsequently transferred (16 V, overnight) onto Immobilon-P PVDF Membranes (Millipore) using the Criterion^TM^ System (BioRad). At least three independent samples per genotype were probed with the following primary antibodies (in 5% milk, overnight at 4 **°**C): RONIN (1:1000, BD Biosciences, #562548), HCFC1 (1:2000, Bethyl, #A301-400A), MMACHC (1:1000, NeuroMabs, #N230/21), GFAP (1:2000, DAKO, #Z0334), UBIQUITIN (1:500, Santa Cruz, #sc-8017), ETC Cocktail [ATP5A, UQCRC2, SDHB, NDUFB8, COX1] (1:1000, Abcam, #ab110413), and ETC Native Blue Cocktail [NDUFA9, SDHA, UQCRC2, ATP5A, COX4] (1:1000, Abcam, #ab110412), LETM1 (1:1000, Proteintech, # 16024-1-AP), and WDR12 (1:1000, Abcam, #ab95070). GAPDH (1:5000, Millipore, #MAB374) and VINCULIN (1:3000, Cell Signaling, #13901) were used as loading controls. The blots were then washed and incubated with HRP-conjugated secondary antibodies from the appropriate species (BioRad) and detected using Clarity Western ECL substrate (BioRad, #170-5061) and imaged using ChemiDoc Touch Imager (BioRad). Relative changes in band intensity were quantified with Image Lab Software (BioRad). The Student’s t-test was used to determine differences between wild type and *Ronin*^*+/F80L*^ or *Ronin*^*F80L/F80L*^, or *Hcfc1*^*A115V/Y*^. *P* values < 0.05 were considered significantly different from controls.

### Co-immunoprecipitation (co-IP)

Embryonic brains, the O9-1 neural crest cell line, or HEK293T cells were homogenized in lysis buffer. Aliquots of clarified protein lysate (500 µg per sample) were diluted in 500 µL lysis buffer supplemented with Halt^TM^ Protease Inhibitor Cocktail (ThermoFisher, #78430), Halt™ Phosphatase Inhibitor Cocktail (ThermoFisher, #78420) and incubated with 1 µg of the following primary antibodies overnight at 4 °C with mixing: RONIN (Bethyl, #A303-180A), HCFC1 (Bethyl, #A301-400A), CARM1 (Bethyl, #A300-421A), C-MYC (Santa Cruz, #sc-40), and ZNF143 (Proteintech, #16618-1-AP) Rabbit IgG (Millipore, #12-370), Mouse IgG (Millipore, #12-371). The next day, the protein/antibody complexes were captured using Protein A/G Magnetic Beads (ThermoFisher, #88802) by incubating for 1 h. at room temperature with rocking. The beads were then washed, and protein eluted with SDS-PAGE reducing sample buffer (50 µL 1× PBS plus 10 µL 6× sample buffer) by boiling for 10 min. Next, 2% of input protein and 15 µL of IP reactions were loaded onto a 4–15% Criterion^TM^ TGX precast midi protein gel (BioRad, #567-1084) and Western blots performed as described above. The following primary antibodies were used in western blot detection of co-immunoprecipitation: RONIN (1:1000, BD Biosciences, #562548), HCFC1 (1:2000, Bethyl, #A301-400A), CARM1 (1:2000, Bethyl, #A300-421A), C-MYC (1:500, Santa Cruz, #sc-40), and ZNF143 (1:800, Proteintech, #16618-1-AP) and detected with the following secondary antibodies (both from Jackson Immuno Research Laboratories): HRP-conjugated mouse anti-rabbit IgG (#211-032-171), and HRP-conjugated goat anti-mouse IgG (#115-035-174).

### Cycloheximide chase

Wild type or *Ronin*^*F80L/F80L*^ MEFs were seeded in 35 mm tissue culture dishes and incubated in 5% CO_2_ overnight in complete culture medium (DMEM, 10% FBS, 1% Pen/Strep). When the cells reached 60–70% confluency, they were incubated with complete medium containing 300 μg/mL cycloheximide (Sigma, #C7698, 10 mL/mL stock in H_2_O). Cells were harvested at different time points (0, 1, 4, 8, or 12 h), washed with cold PBS, lysed, and processed for protein extraction in lysis buffer supplemented with Halt^TM^ Protease Inhibitor Cocktail (ThermoFisher, #78430) and Halt™ Phosphatase Inhibitor Cocktail (ThermoFisher, #78420). Western blot analysis was performed using anti-RONIN (1:1000, BD Biosciences, # 564528) and anti-VINCULIN (1:3000, Cell Signaling, #13901) primary antibodies, and HRP-conjugated goat, anti-Mouse and goat, anti-Rabbit IgG secondary antibodies (1:3000, BioRad, #170-6516 and #170-6515).

### Puromycin assay

Wild type or *Ronin*^*F80L/F80L*^ MEFs were cultured in growth medium (DMEM, supplemented with 10% FBS and Pen/Strep). Puromycin (Sigma, # P8833) was then added to a final concentration of 10 μg/ml and incubated at 37 °C for 10 min. Cells were then washed with cold PBS three times and harvested. Freshly dissected E16.5 wild type or *Ronin*^*F80L/F80L*^ mouse brains were isolated in cold PBS, chopped very finely on ice and were then incubated in DMEM containing 10% FBS, 1% Pen/Strep and puromycin (final concentration of 10 μg/ml) for 30 min at 37 °C. Cells were then washed with cold PBS three times and harvested. For both MEFs and brains, 30 μg of cell lysate was loaded onto a 4–15% Criterion^TM^ TGX Stain-Free precast gel (BioRad, #5678084) for electrophoresis and subsequently imaged using ChemiDoc Touch Imageer (BioRad) for total protein loading. Gels were then transferred onto PVDF membranes and analyzed by Western blot using anti-Puromycin antibody (1:1000, Karafast, #EQ0001) and HRP-conjugated goat, anti-Mouse IgG (1:3000, BioRad, #170-5616). Relative changes in puromycin labeling and stain-free loading control intensity were quantified using ImageJ with the gel analysis function.

### Polysome profiling

Polysome profiling was done in accordance with standard procedure. Briefly, a 10–50% sucrose gradient was poured using gradient buffer of 20 mM HEPES (pH 7.5), 5 mM MgCl_2_, 100 mM KCl and 2 mM DTT as the base solution. E18.5 brains were dissected out in cold PBS and placed in 200 μl RNA*later* RNA stabilization buffer (Qiagen, #10107980). Brain lysates were prepared in gradient buffer supplemented with 1% Triton X-100, 0.5% sodium deoxycholate, 200 U/ml RNase, and 100 ug/ml cycloheximide. Brains were homogenized with 50 strokes of a Teflon-tipped Dounce followed by 10 passes through a 28-gauge needle. Lysates were then precleared at 16,000 × *g* for 10 min prior to quantification of total RNA with a nanodrop spectrometer and loading of equal amounts of RNA onto the sucrose gradient. Gradients were run at ~217,500 × *g* in a SW-40 rotor at 4 °C for 2 h. Gradients were fractionated in an ISCO gradient fractionator with UV monitoring and data was collected using Logger Lite software (Vernier). For quantification of the polysome traces, the area under the curve was calculated for each peak of each sample using PRISM v8 software (GraphPad).

### SunRiSE assay

SunRiSE (SUnSET-based Ribosome Speed of Elongation) analysis was performed as previously described^50^. Briefly, wild type or *Ronin*^*F80L/F80L*^ MEFs were seeded in 60-mm tissue culture dishes in growth medium (DMEM, supplemented with 10% FBS and Pen/Strep), and incubated at 37 °C with 5% CO_2_ overnight to 60–70% confluence. The cells were then treated with 2 μg/ml of harringtonine (Abcam, #ab141941) for different time points (0, 0.5, 1, 2, 3, 5, and 10 min) followed by puromycin (Sigma, # P8833) treatment (10 μg/ml at 37 °C for 10 min). Cells were then washed with ice-cold PBS three times and harvested for Western blots performed as described above with anti-Puromycin antibody (1:1000, Karafast, # EQ0001).

### RNA extraction and quantitative rtPCR

Embryonic brains were dissected, and total RNA immediately purified using the RNeasy Mini Kit (Qiagen). The purified RNA was reverse transcribed using the SuperScript III first strand synthesis kit with Oligo(dT)_20_ and random hexamer priming (Invitrogen). The Taqman^®^ gene expression assay from Applied Biosystems was used for qrtPCR and the following probes from Applied Biosystems were used to detect gene expression: *Mmachc* (Mm00482455_m1), *Ronin* (Mm01250553_s1), *Hcfc1* (Mm00468501), *Nestin* (Mm00450205), *Vimentin* (Mm01333430), *Musashi-1* (Mm01203522), *Sox2* (Mm00488369), *Tbr2* (Mm01351984), *Cux2* (Mm00500377), *Ctip2* (Mm07297701), *Brn2* (Mm00843777), *Gfap* (Mm01253033), *Slc1a3* (Mm00600697), *Aqp4* (Mm00802131), and *S100b* (Mm00485897). The following primers were used for qrtPCR using the SYBR^TM^ Green Master Mix from Applied Biosystems (#4309155): Rps3_F: 5′-GCTGAAGATGGCTACTCTGG-3′, Rps3_R: 5′-ACAACTGCGGTCAACTCTC -3′, Rps13_F: 5′-CTTGATTTCCTGTGCCGTTTC-3′, Rps13_R: 5′-AGACGTCAACTTCAGCCAC-3′, Rpl6_F: 5′-AGAGTGGTTTTCCTGAAGCAG-3′, Rpl6_R: 5′-TGCTTGTGACTGGGCCTCTTGT-3′, Rpl8_F: 5′-CCCGTCTATTTCCTCTTTCG-3′, Rpl8_R: 5′-GAAGTCCACAGCACGTAGG-3′, Mrps22_F: 5′-TCCTCAATCTCTGTGTTGCC-3′, Mrpls22_R: 5′-CAAAATACCAAGCCATTCCACC-3′, Mrps25_F: 5′-AGGAGATCATGGAGCACATAAAG-3′, Mrps25_R: 5′-GGCCTTCTACTTCGCACATG-3′, Mrpl42_F: 5′-GCTTTGTCTAGTGCTTGTCAC-3′, Mrpl42_R: 5′-ACTTTGGTGTGTTCGTAGGG-3′, Mrpl54_F: 5′-ACTCAGCTCACCACACATG-3′, and Mrpl54_R: 5′-TCGGGACTCTGGTTCTAGTTC-3′.

QrtPCR was performed on a StepOnePlus Real-Time PCR System (Life Technologies) under the following conditions: 50 °C for 2 min, 95 °C for 10 min, 40 cycles of 95 °C for 15 s and 60 °C for 1 min. To determine the relative quantification of gene expression, qrtPCR was performed in triplicate for each independent control and mutant cDNA sample (a minimum of three independent samples per genotype were compared). Mean Δ*C*t values for each gene were normalized against the housekeeping genes *Gapdh* (Mm4352932)*, Pgk1* (Mm00435617), and *Beta-actin* (Mm4352933) and corresponding ΔΔ*C*t values were log2-transformed to obtain fold change values. For data analysis, the Pfaffl method was used to determine relative gene expression ratios and a *p* value of <0.05 was considered significant^85^.

### RNA-seq analysis

Three independent E16.5 brains per genotype were disected in ice-cold 1× PBS and total RNA was extracted using the RNeasy Mini Kit (Qiagen). Next, 1 µg of total RNA was incubated with oligo(dT) magnetic beads (SeraMag) in order to enrich for mRNA with poly-A tails. The eluted RNA was incubated at 94 °C in Tris buffer with potassium acetate and magnesium acetate resulting in fractionated RNA (200–500 nucleotide range). This poly-A-selected RNA was ethanol precipitated with sodium acetate, then resuspended in water. cDNA was generated using the Invitrogen SuperScript III reverse transcription kit containing random oligonucleotide hexamers. Next, the RNA was degraded by addition of RNase and DNA polymerase was added to generate a second strand. The resulting DNA was then used for standard Illumina adapter ligation for sequencing as described in previously^86^. To generate blunt-ended DNA molecules, the 5′ and 3′ overhangs were filled-in by T4 polymerase and T4 polynucleotide kinase. Next, the Klenow fragment (exo−) was used to add a deoxyadenosine (dA) 5′ tail to the DNA strands. Using T4 ligase, double stranded DNA adapters with 3′ thymidine overhangs were ligated to the dA-tailed library as previously described^87^.

Alignment of RNA-Seq data was performed using STAR (version 2.7.3a)^88^. All reads were mapped to the mm9 genome. Genes differentially expressed between the control and mutant samples were detected using ‘DESeq’ version 2.13. Significantly differentially expressed genes were defined using cutoffs of *p*-value ≤ 0.05; fold change ≥ 1.5 and FDR ≤ 0.10. A total of 980 genes that were upregulated and 1301 that were downregulated in *Ronin*^*F80L/F80L*^ brains relative to control. All GO aalysis was perfomred with Metascape^89^.

### Chromatin immunoprecipitation (ChIP) and ChIP-qPCR

For all ChIP experiments (ChIP-seq and ChIP-qPCR), freshly harvested E16.5 brains from wild type or *Ronin*^*F80L/F80L*^ mice were cut into small pieces and the tissue cross-linked for 15 min and chromatin isolated using the ChIP-IT High-Sensitivity Kit (Active Motif, #53040). This was followed by sonication (25% amplitude pulse for 30 s on and 30 s off) for a total sonication on-time of 5 min 30 μg of sheared chromatin was then subjected to immunoprecipitation (overnight at 4 °C) using a mouse, anti-RONIN (BD Biosciences, #562548), or mouse, anti-IgG antibody (Millipore, #12-371) following the kit protocol. The reactions were then incubated with pre-washed Protein G beads on a rotator at 4 °C for 3 h. The captured, antibody-bound protein/DNA complexes were then washed in the supplied filtration columns and chromatin eluted from the column. The chromatin cross-linking was then reversed and the DNA purified and eluted from the supplied columns. The purified DNA was then analyzed by qPCR using the SYBR Green Master Mix (ThermoFisher, #4309155) and the ViiA 7 Real-Time PCR system (Applied Biosystems). As a positive control, we used RnBSF 5′-AGGACGAGCTTCATCTGGAA-3′ and RnBSR 5′-CCTGGAAAGGAGGACTACGC-3′ to amplify a previously-identified RONIN auto-regulatory binding site within the *Ronin* promoter^76^. As a negative control, we used NRnBSF 5′-CAGCCCTCAGTTCTGGAAAG-3′ and NRnBSR 5′-GCTGACCATGTGGCCTATCT-3′, which amplify a region of the *Ronin* gene not bound by RONIN^76^. Cycle conditions: 50 °C/2 min, 95 °C/10 min, 40 cycles of [95 °C/15 s, 60 °C/1 min]. To determine the RONIN binding ability on *Mmachc* promoter, E16.5 brains from wild type and *Ronin*^*F80L/F80L*^ mice were harvested and prepared similarly. We used MmcRBSF 5′-GGACAAAAGTAACTACGTGTACCA-3′ and MmcRBSR 5′-AACGCACGCTGAACGCT-3′ primers to aimplify the promoter region of *Mmachc* bound by RONIN.

### ChIP-seq analysis

Freshly harvested E16.5 brains from CD-1 mice were cut into small pieces and the tissue cross-linked for 15 min and chromatin isolated using the ChIP-IT High-Sensitivity Kit (Active Motif, #53040). We thank the Duke University School of Medicine for the use of the Sequencing and Genomic Technologies Shared Resource, which provided ChIP-seq library generation and sequencing services. All libraries were sequenced on an Illumina Hiseq 2500.

Reads were mapped to the mm9 assembly (NCBI Build 37) using bowtie2 (version 2.3.4.2)^90^. The ChIP-Seq signal was normalized to a 10 million read total and visualized in the UCSC genome browser after tag directories were generated using HOMER (version v4.10.3)^91^. ChIP enriched peaks were identified using HOMER with default transcription factor settings (findPeaks-style factor). All Ronin ChIP-seq peaks were called using input ChIP-seq data as background, generating 33,394 peaks with a peak score greater than 1.95. The nearest genes associated to the enriched peaks were annotated using the annotatePeaks function and de novo motif discovery was performed using the findMotifsGenome function in HOMER. Genes associated to ChIP-Seq peaks were overlaid with differentially expressed genes in the *Ronin*^*F80L/F80L*^ versus wild type RNA-seq dataset. The overlaid gene list was analyzed with Metascape for enriched gene ontology terms.

### Single-run proteomic profiling

Proteomic profiling was performed on independent MEF lines derived from three individual wild type and three individual *Ronin*^*F80L/F80L*^ embryos. Cell pellets were lysed by freeze-thaw and sonication in 200 μL ABC buffer (50 mM ammonium bicarbonate, 1 mM CaCl_2_). For each sample, 50 μg of protein was subjected to proteolysis by endoproteinase Lys-C (Wako, #129-02541) at room temperature for 2 h. Proteins were further digested with trypsin (Promega, #V5113) at 37 °C for 16 h. Digestion was stopped by the addition of 1% formic acid (Sigma, #56302). Peptides were extracted with 80% acetonitrile in 0.1% formic acid and the precipitate was removed by centrifugation. Peptide concentration was measured using the Peptide Assay Reagent Kit (Pierce, #23275) using a DS-11 Spectrophotometer (DeNovix). Peptides were aliquoted and dried in a speed vacuum centrifuge. Lyophilized peptides were dissolved in 5% MeOH/0.1% formic acid.

Peptides were analyzed using a nano-LC 1200 system (Thermo Fisher Scientific) coupled to Orbitrap Fusion (Thermo Fisher Scientific) mass spectrometer (MS) using a 75 min discontinuous gradient of 4–26% acetonitrile/0.1% formic acid followed by a 5 min wash of 90% acetonitrile at a flow rate of 800 nL/min with a 5 cm column, or a 130 min discontinuous gradient of 4–28% acetonitrile/0.1% formic acid followed by a 5 min wash of 90% acetonitrile at a flow rate of 200 nL/min with a 20 cm column. The full MS scan was performed in Orbitrap analyzer in the range of 300–1400 *m/z* at 120,000 resolution followed by an IonTrap HCD-MS2 fragmentation of the top 50 strongest ions with precursor isolation window of 2 *m/z*, collision energy 32%, AGC of 5000, maximum injection time of 50 ms or 30 ms, respectively.

Proteome Discoverer (PD version 2.0.0.802; Thermo Fisher Scientific) was used to process raw files, with database searches performed with the Mascot search engine (v2.5.1, Matrix Science). Peptides in a 350–10,000 mass range were generated in silico from mouse RefProtDB protein database (2020-03-24 download) with trypsin (without cleavage before P) and up to 2 missed cleavages. Precursor mass tolerance was set at 20 ppm, and a fragment mass tolerance of 0.5 Da. We allowed the following dynamic modifications: acetyl (Protein N-term), oxidation (M), carbamidomethyl (C), DeStreak (C), and deamidated (NQ). peptide-spectral matches (PSMs) were validated with Percolator (v2.05)^92^. The target strict and relaxed FDR levels for Percolator were set at 0.01 and 0.05 (1 and 5%), respectively. Label free quantification of PSMs was made using Proteome Discoverer’s Area Detector Module. We used gpGrouper (v1.0.040) for gene product inference and label-free iBAQ quantification with shared peptide distribution^93^. We then median-normalized iBAQ values by sample for downstream analyses. We employed the moderated *t*-test to calculate *p*-values and log2 fold changes for differentially expressed proteins as implemented in the R package limma^94^. Multiple-hypothesis testing correction was performed with the Benjamini–Hochberg procedure^95^. Finally, among the differentially expressed proteins (*p* < 0.05), we performed functional annotation clustering using The Database for Annotation, Visualization, and Integrated Discovery (DAVID) v6.8.

### TMT10 protein profiling of brain tissue

The mouse brain tissue was pulverized into powder and protein extraction was carried out in 8 M urea followed by alkylation, reduction, and enzyme digestion. Briefly, the protein lysate in 8 M urea was alkylated using 10 mM iodoacetamide, diluted to 2 M urea, digested with LysC (Wako 129 02541) for 2 h at room temperature and Trypsin (GenDepot T9600) overnight at 37  °C. Peptides were desalted on a Sep-Pak C18 cartridges (Waters, 50 mg 1cc). 50 µg of desalted peptides were dissolved in 50 mM HEPES (pH8.5) and labeled with TMT10 plex reagent as per manufacturer’s protocol in a 1:8 peptide to TMT ratio (Thermo Scientific, 90110). After the labeling efficiency check, samples were pooled, desalted and separated using an off-line high pH reversed-phase column into 96 fractions concatenated into 24 pools as previously described^96^.

The off-line fractionated peptide pools were separated on an online nanoflow EASY-nLC1200 UHPLC system (Thermo Fisher Scientific) and analyzed on Orbitrap Fusion Lumos ETD mass spectrometer (Thermo Fisher Scientific). For each fraction, we loaded 1 µg peptide on a 20 cm 75 µm ID column (1.9 µm Reprosil-Pur Basic C18, Dr. Maisch GmbH, Germany) using a 93 min gradient of 2–60% acetonitrile in 0.1% formic acid at 200 nl/min followed by a 90–50% acetonitrile wash for 17 min The data dependent acquisition was done using Full MS-ddMS2 at high resolution in Orbitrap. Full MS resolution was set at 120,000 for scan range 300–1400 *m/z*, AGC 5e5, and maximum injection time (IT) 50 ms. For MS2 spectra, the AGC was set at 1e5 with 105 ms IT, 50,000 resolution, isolation window of 0.7 *m/z*, and normalized collision energy 38%. The cycle time was set to 2 s with dynamic exclusion of 20 s.

The TMT data was searched in Proteome Discoverer (PD 2.1; Thermo Fisher Scientific) using Mascot search engine v2.4, (Matrix science) against mouse RefProtDB protein database (2020-03-24 download). Searches were performed using a 20 ppm precursor ion tolerance and 0.02 Da product ion tolerance. TMT on lysine residues and peptide N termini (+229.1629 Da) and carbamidomethylation of cysteine residues (+57.0215 Da) were set as static modifications (except when testing for labeling efficiency, in which the TMT modifications were set to variable), while oxidation of methionine residues (+15.9949 Da) was set as a variable modification. Reporter ion intensities were adjusted to correct for the isotopic impurities according to the manufacturer’s specifications. The peptide spectral matches (PSMs) output file from PD was exported, and the gene product inference and isobaric-based iBAQ quantification was carried by gpGrouper algorithm^93^. We median-normalized iBAQ values by sample for downstream analyses. We employed the moderated *t*-test to calculate *p*-values and log2 fold changes for differentially expressed proteins as implemented in the R package limma^94^. Multiple-hypothesis testing correction was performed with the Benjamini–Hochberg procedure^95^. Among the differentially expressed proteins (*p* < 0.05), we performed functional annotation clustering using The Database for Annotation, Visualization, and Integrated Discovery (DAVID) v6.8.

### Statistics and reproducibility

All utilized statistical tests are described in the figure legends. All experiments were repeated a minimum of three times with similar results.

### Reporting summary

Further information on research design is available in the Nature Research Reporting Summary linked to this article.

## Supplementary information

Supplementary Information

Description of Additional Supplementary Files

Supplementary Dataset 1–16

Reporting summary

## Data Availability

RNA-seq and ChIP-seq raw data files are deposited in the Gene Expression Omnibus (GEO) database (GSE161763). Mass spectrometry raw data files are deposited at ProteomeXchange (PXD0022310), ftp://massive.ucsd.edu/MSV000086402. All data generated and/or analyzed during the current study are available from the corresponding author on reasonable request. Source data are provided with this paper.

## Source data

Source data
