# Supplementary material for: Mutations in Hcfc1 and Ronin result in an inborn error of cobalamin metabolism and ribosomopathy
Source: Nat Commun. 2022 Jan 10;13:134. doi: 10.1038/s41467-021-27759-7 (PMC8748873; doi:10.1038/s41467-021-27759-7)
Supplement: Supplementary file 1 — Supplementary Information [file 41467_2021_27759_MOESM1_ESM.pdf]

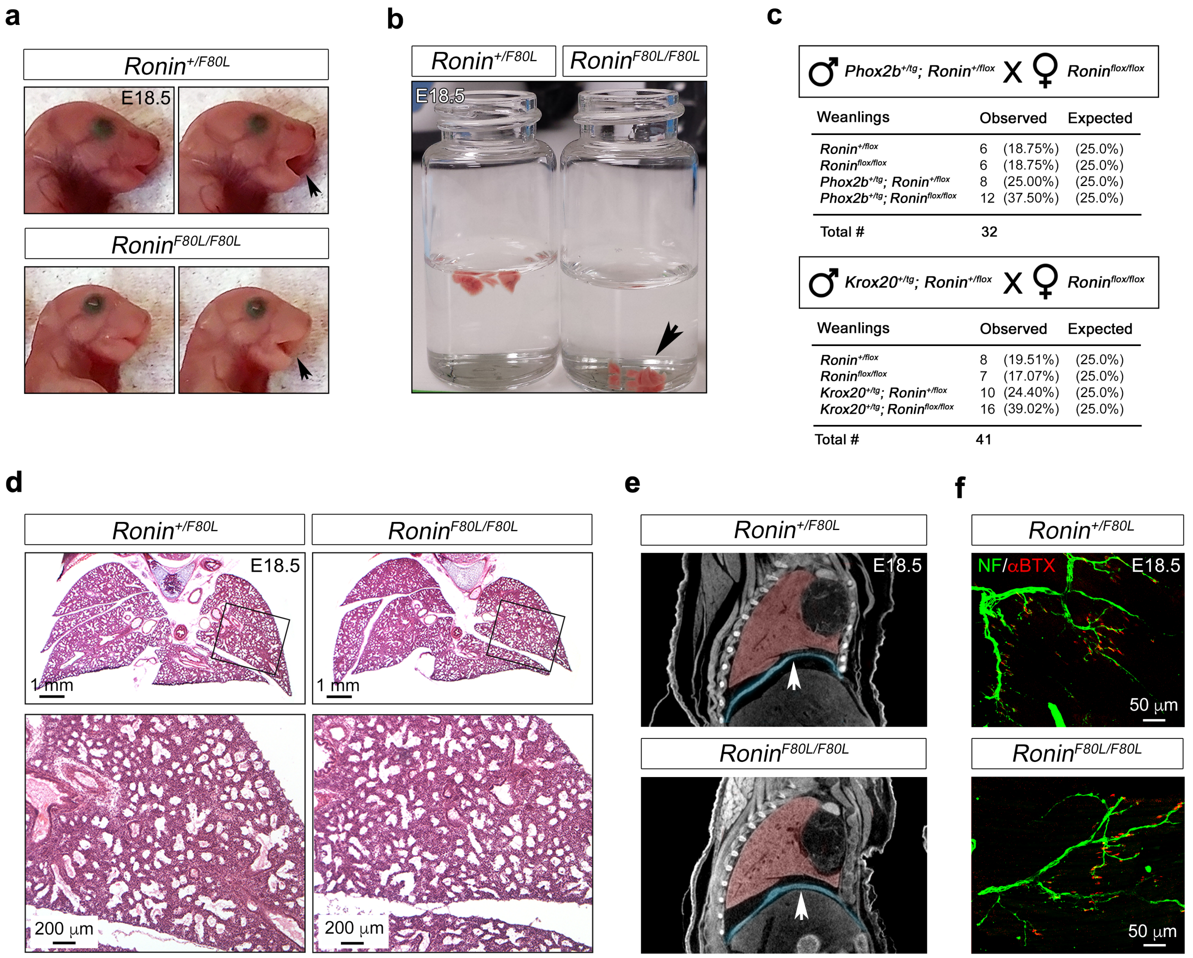

Supplementary Figure 1. *$Ronin^{F80L/F80L}$* mice cannot breathe, but this does not appear to be due to any obvious defect of the brainstem, lungs, or diaphragm. As indicated by gasping motions, C-sectioned E18.5 *$Ronin^{F80L/F80L}$* pups were alive and maintained the drive to breathe (a). However, within minutes, 100% the homozygous mutant pups became cyanotic and died. Lungs were immediately isolated from these pups and placed in a vial of water. While the heterozygous lungs floated to the surface, the homozygous lungs sank indicated that they were unable to be filled with air (b). The fact that the *$Ronin^{F80L/F80L}$* pups exhibited the drive to breathe, yet could not inflate their lungs with air, argued against a defect within the neuronal breathing centers of the hindbrain. This conclusion was further supported by the finding that *Krox20-Cre* and *Phox2b-Cre* mediated brain stem CKOs of *Ronin* are viable at weaning (c)[1,2]. However, hematoxylin and eosin (H&E) staining of lung cryosections showed structure and patterning indistinguishable from controls (d). Micro-CT assessment of diaphragm structure and positioning appeared normal (e). Alpha-bungarotoxin staining and neurofilament immunofluorescent assess of diaphragm innervation also appeared grossly normal (f). In total, these data suggest the inability of the *$Ronin^{F80L/F80L}$* neonates to breathe may not be due to defects in the CNS, lungs, or diaphragm. $N \geq 3$ biologically independent samples per genotype.

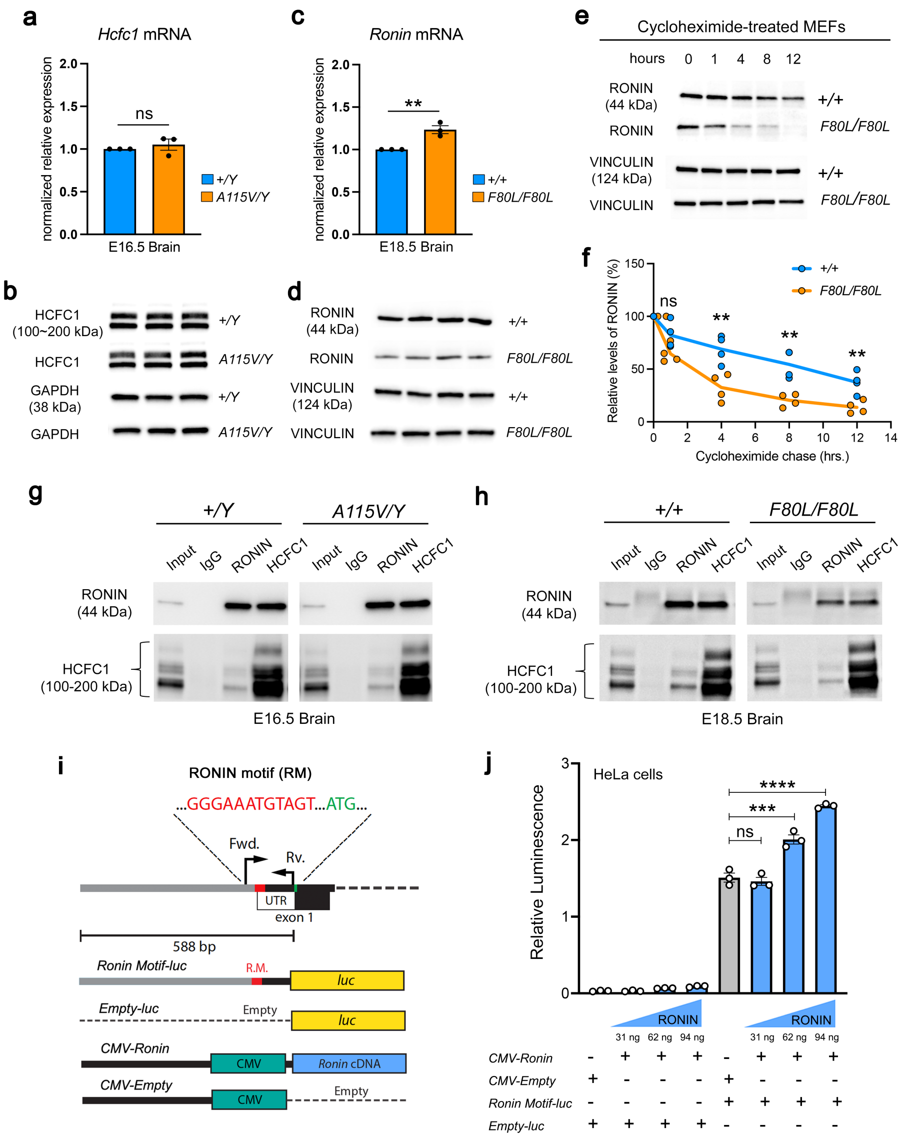

Supplementary Figure 2. RONIN transcriptionally activates *Mmachc* and the *Ronin F80L* mutation causes instability of RONIN protein without affecting its interaction with HCFC1. QrtPCR analysis of E16.5 brains revealed that *Hcfc1* mRNA expression is not affected by the *A115V* hemizygous mutation (a). Western blot analysis of E16.5 *Hcfc1*$^{A115V/Y}$ brain lysates showed that the HCFC1 protein level is also unchanged (b). The *Ronin* mRNA expression level is increased in *Ronin*$^{F80L/F80L}$ E18.5 brains and is consistent with previous ChIP-seq data suggesting RONIN undergoes transcriptional autoregulation (c)[3,4]. However, despite the increase in *Ronin* mRNA, Western blots showed reduced RONIN protein levels in *Ronin*$^{F80L/F80L}$ E18.5 brains (d). Through cycloheximide chase assessment of protein stability, we showed that the RONIN F80L protein degrades faster in *Ronin*$^{F80L/F80L}$ mutant MEFs versus wild type (e and f). RONIN/HCFC1 co-immunoprecipitation experiments on *Hcfc1*$^{A115V/Y}$ and *Ronin*$^{F80L/F80L}$ MEFs showed that the mutant proteins were still capable of binding one another (g and h). We cloned a 588 bp region upstream of the mouse *Mmachc* start codon that includes a RONIN binding motif (R.M.). This fragment was then inserted into a luciferase reporter plasmid *(Ronin Motif-luc)* that was transfected into HeLa cells (i). To overexpress *Ronin*, we also cloned a *Ronin* cDNA driven by a CMV promoter (i). The luciferase assay suggested that endogenous RONIN in HeLa cells is likely capable of driving luciferase reporter expression (j, gray bar). By transfecting the mouse *Ronin* cDNA, this activity was increased in a dosage-dependent manner (j, blue bars). The data in a, c, and f are shown as the mean ± SEM and n ≥ 3 biologically independent samples per genotype. Statistically significant differences between genotypes were determined using the *t* test (two-tailed). $^{**}p < 0.01$. The data in j are shown as the mean ± SEM and n = 3 biologically independent samples per treatment. Statistically significant differences between genotypes were determined using ANOVA and Dunnett's multiple comparisons test. $^{***}p<0.001$, $^{****}p<0.0001$. Source data are provided as a Source Data file.

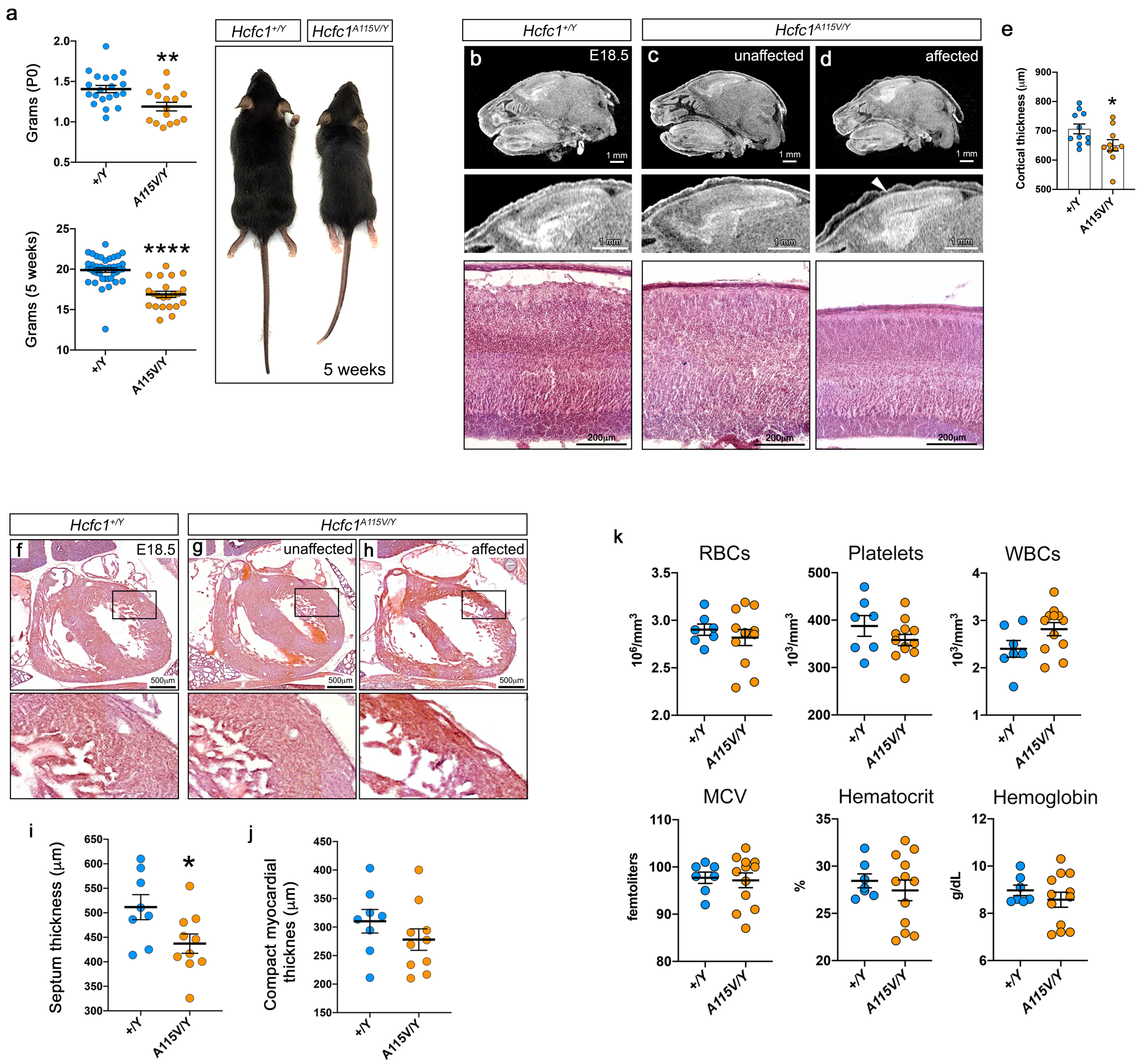

Supplementary Figure 3. Partially penetrant phenotypes in *Hcfc1*$^{A115V/Y}$ mice. At birth, the *Hcfc1*$^{A115V/Y}$ mice had a lower body weight than wild type and this persisted into adulthood (a). Micro-CT imaging of E18.5 *Hcfc1*$^{A115V/Y}$ brains revealed that a subset of mutants was indistinguishable from wild type while some exhibited mild cortical thinning (d, arrowhead). Quantification of cortical thickness from H&E cryosections confirmed that, while variable, the average thickness of the *Hcfc1*$^{A115V/Y}$ cortex is reduced (b-e). H&E staining of E18.5 heart cryosections revealed ventricular hypoplasia that was also partially penetrant with some *Hcfc1*$^{A115V/Y}$ appearing unaffected (f-h). Quantification revealed that the average thickness of the *Hcfc1*$^{A115V/Y}$ septum myocardium was reduced (i) while the average myocardial thickness was unchanged (j). Analysis of plasma showed that red blood cells, hemoglobin, and hematocrit levels were reduced in a few *Hcfc1*$^{A115V/Y}$ mice, but on average, hematology results were unchanged (k). All data are shown as mean ± SEM and $n \geq 3$ biologically independent samples per genotype. Statistically significant differences between genotypes were determined using the *t* test (two-tailed). *p<0.05, **p<0.01, ***p<0.001, ****p<0.0001. Source data are provided as a Source Data file.

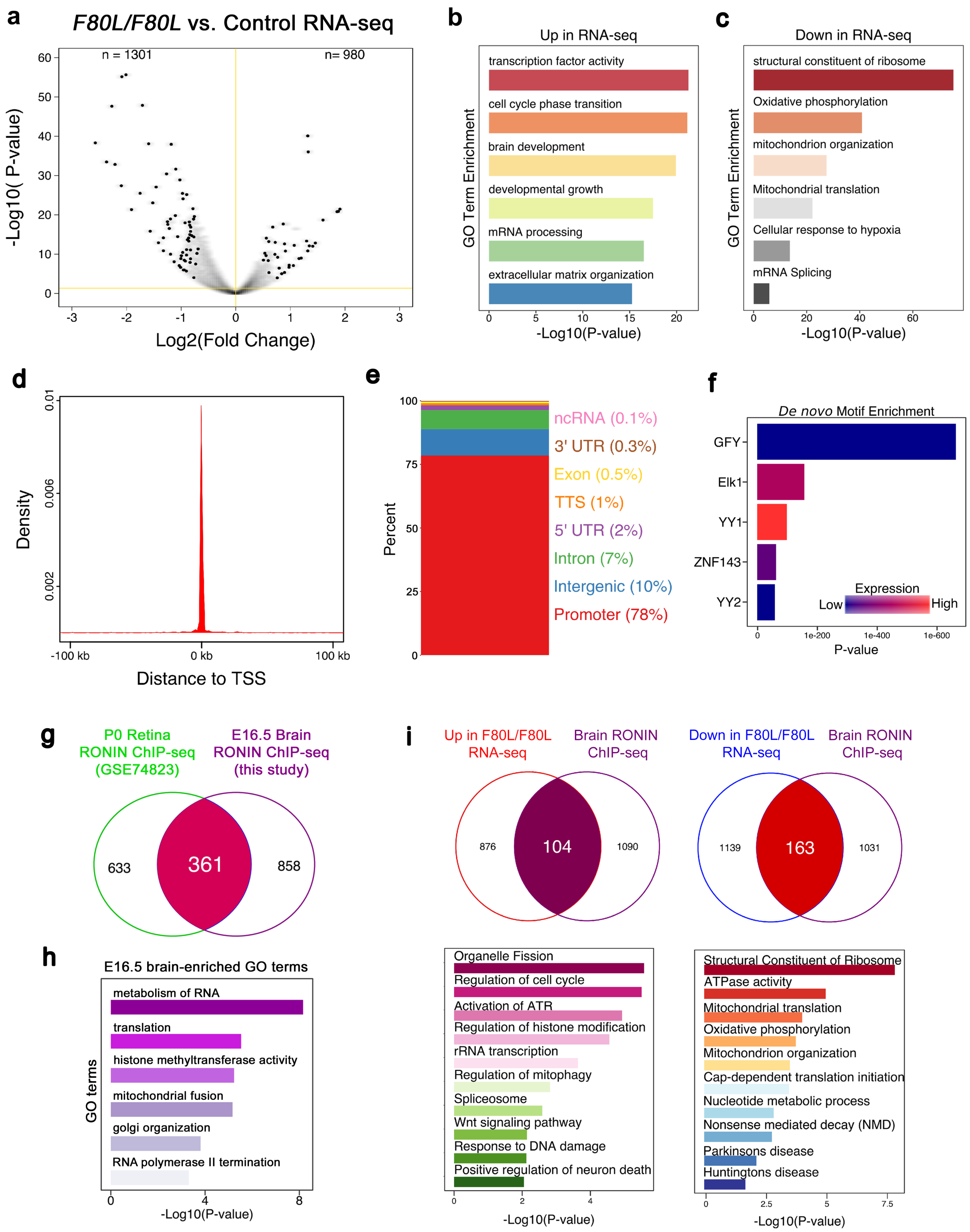

Supplementary Figure 4. RONIN directly regulates the expression of genes encoding ribosomal subunits. Volcano plot of the *Ronin*$^{F80L/F80L}$ RNA-seq data showed 980 genes were significantly upregulated and 1,301 genes were significantly downregulated in E16.5 *Ronin*$^{F80L/F80L}$ brains (0.5 log2 fold change and adjusted p-value < 0.05) (a). GO analysis of the genes upregulated and downregulated in the RNA-seq showing that the most enriched GO term on the downregulated side was "structural constituents of the ribosome" (b-c). RONIN ChIP-seq peak distribution (d) and site annotation (e) across the E16.5 brain genome showing significant enrichment at promoter regions. De novo motif enrichment analysis of RONIN brain ChIP-seq peaks overlaid with our RNA-seq expression data (average of normalized controls) showing the expected enrichment of the known RONIN motif (GFY). Motifs for Elk1, YY1, ZNF143, and YY2 were also enriched (f). Overlap of RONIN E16.5 brain ChIP-seq with P0 retina ChIP -seq (GSE74823) identified 858/1,219 as being specific to the brain dataset (g) and showed enrichment for the GO terms "metabolism of RNA" and "translation" (h). Overlap of E16.5 brain RONIN ChIP-seq with E16.5 *Ronin*$^{F80L/F80L}$ RNA-seq data with GO analysis (i).

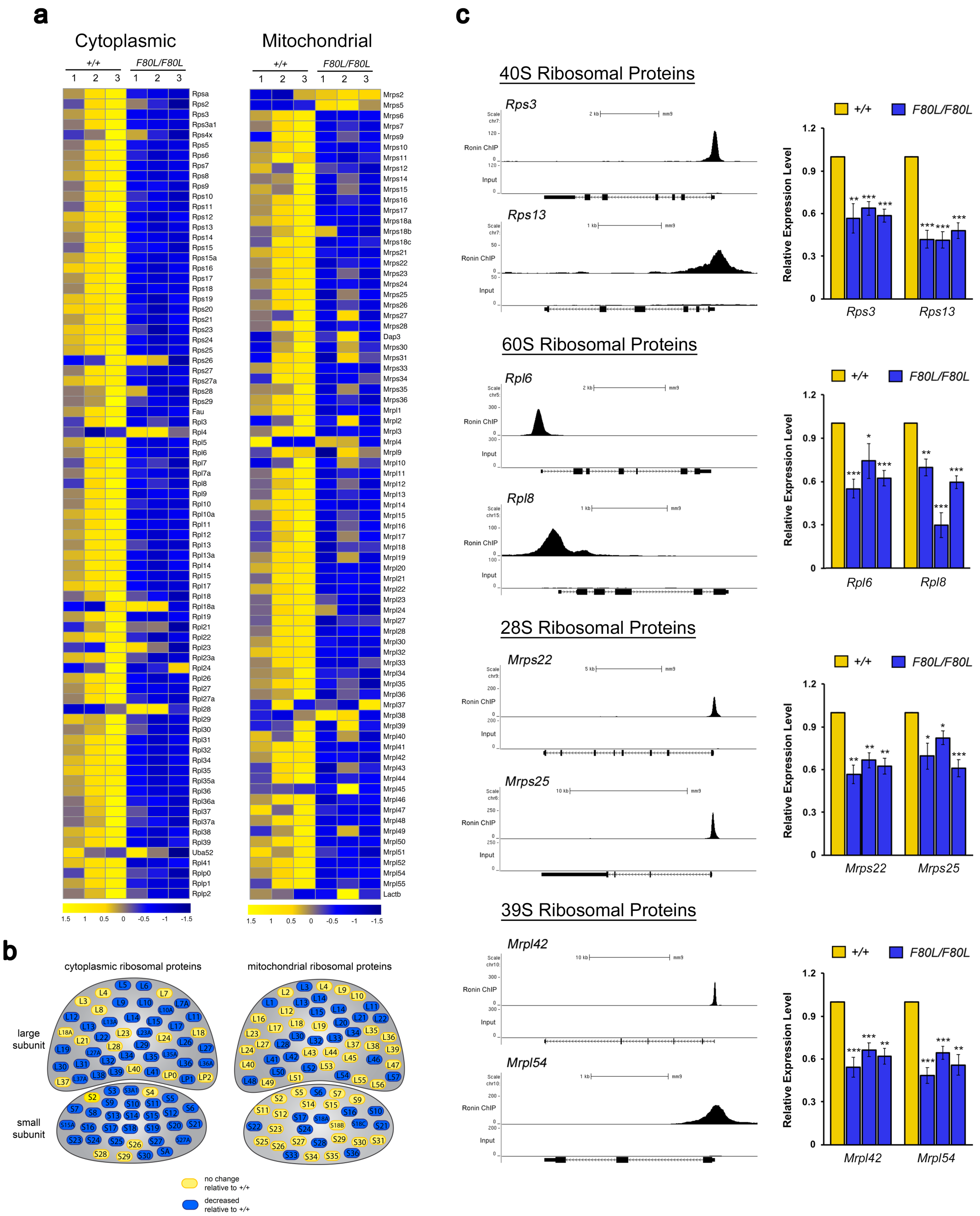

Supplementary Figure 5. A large cohort of genes encoding ribosomal subunits are transcriptionally regulated by RONIN. Heatmap from the *Ronin*$^{F80L/F80L}$ E16.5 brain RNA-seq data showing a reduction in expression of cytoplasmic and mitochondrial ribosomal subunit genes (a). Schematic of the cytoplasmic and mitochondrial ribosomes indicating the subunit genes reduced (blue) or unchanged (yellow) in the *Ronin*$^{F80L/F80L}$ RNA-seq dataset (b). Images of RONIN ChIP-seq peaks within promoter regions for a subset of ribosomal genes representing 40S (small cytoplasmic), 60S (large cytoplasmic), 28S (small mitochondrial), 39S (large mitochondrial) ribosome subunits and qrtPCR analysis validating their reduction in the RNA-seq dataset (c). The data in c are shown as mean ± SEM and n = 3 biologically independent samples per genotype. Statistically significant differences between genotypes were determined using the *t* test (two-tailed). *p<0.05, **p<0.01, ***p<0.001. Source data are provided as a Source Data file.

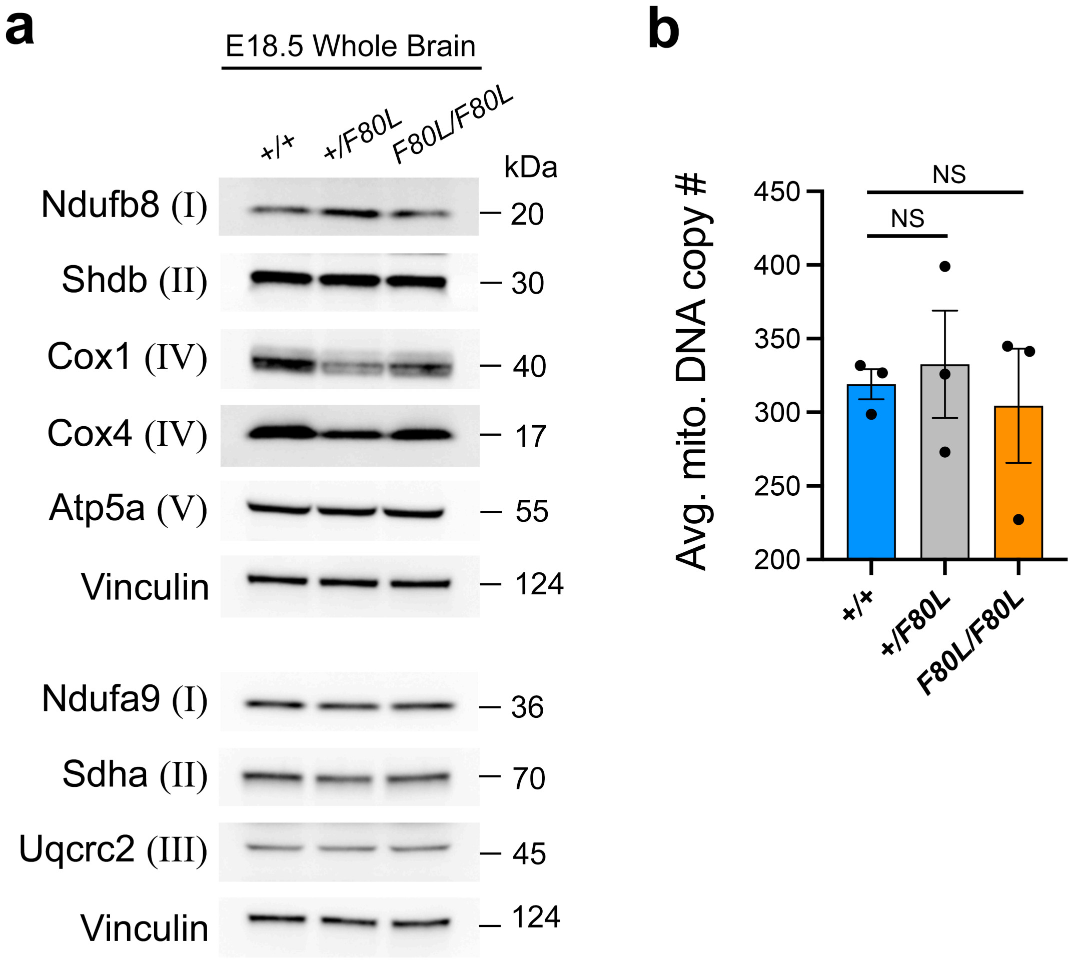

Supplementary Figure 6. *Ronin$^{F80L/F80L}$* mutants do not exhibit mitochondrial defects that were observed in *Ronin* conditional knockout retinae. Western blots of protein subunits within the electron transport chain showed no change on the E18.5 *Ronin$^{F80L/F80L}$* brain as compared to wild type (a). PCR analysis showing the average mitochondria DNA copy number was not altered in *Ronin$^{F80L/F80L}$* and suggests normal mitochondrial content (b). The data in b are shown as mean ± SEM, n = 3 biologically independent samples per genotype. Statistically significant differences between genotypes were determined using ANOVA and Tukey's multiple comparisons test. Source data are provided as a Source Data file.

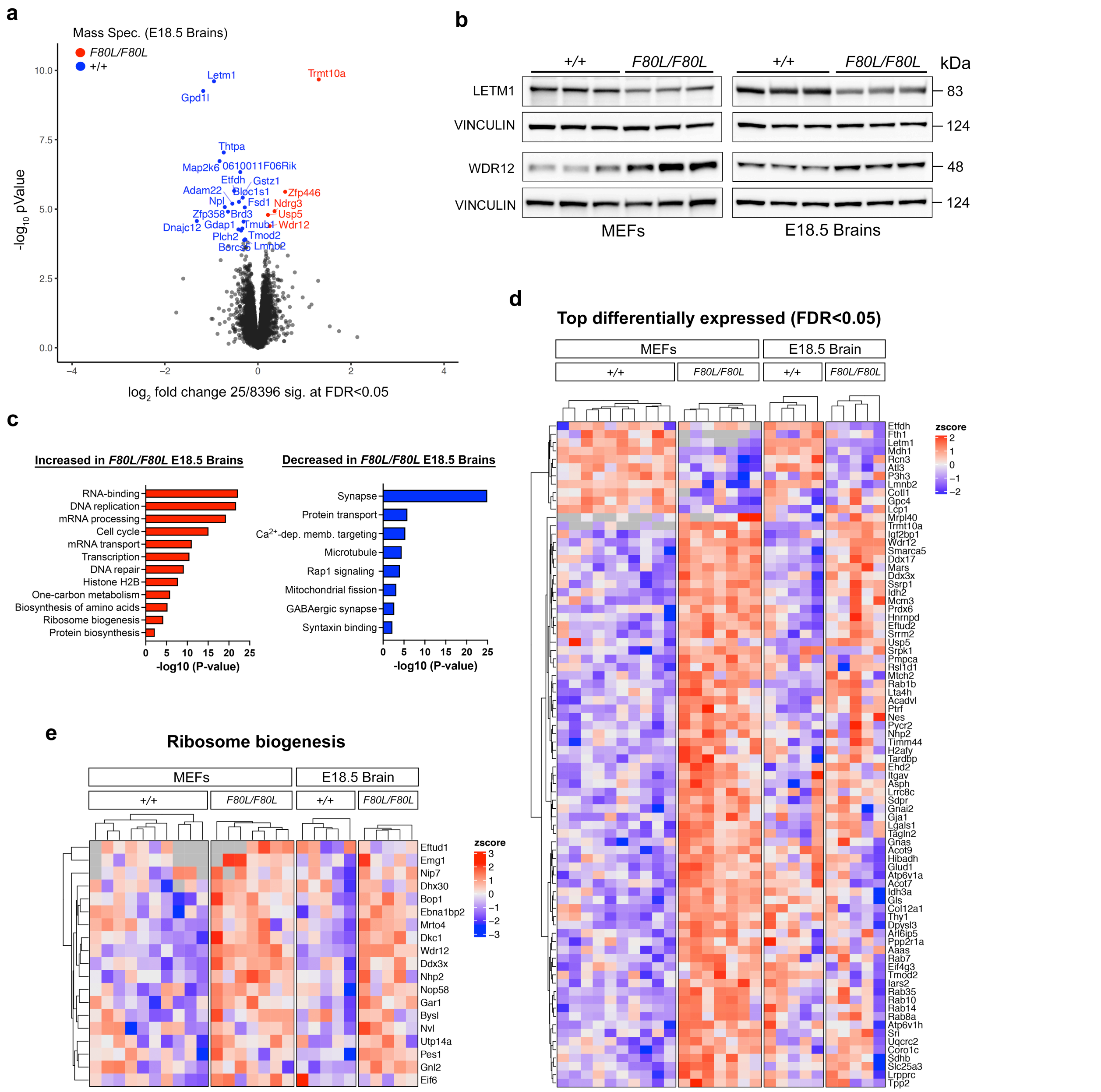

Supplementary Figure 7. *Ronin*$^{F80L/F80L}$ E18.5 brain mass spectrometry shows similar alteration as the mutant MEFs. Volcano plot of the *Ronin*$^{F80L/F80L}$ versus wild type brain protein mass spec. (a). Western blot validation of 2 mass spec. targets (LETM1 and WDR12) that were differentially expressed in both the *Ronin*$^{F80L/F80L}$ MEFs and brains (b). GO analysis of the proteins upregulated and downregulated in the *Ronin*$^{F80L/F80L}$ brain mass spec. (c). Note that, analogous to the mutants MEFs (Fig. 7), the brain also shows an enrichment for proteins involved in ribosome biogenesis and protein biosynthesis. Heat map of the top differentially expressed proteins in common between the *Ronin*$^{F80L/F80L}$ MEFs and brains (d). Heat map of the differentially expressed proteins associated with ribosome biogenesis in common between the *Ronin*$^{F80L/F80L}$ MEFs and brains (e). N ≥ 3 biologically independent samples per genotype. Source data are provided as a Source Data file.

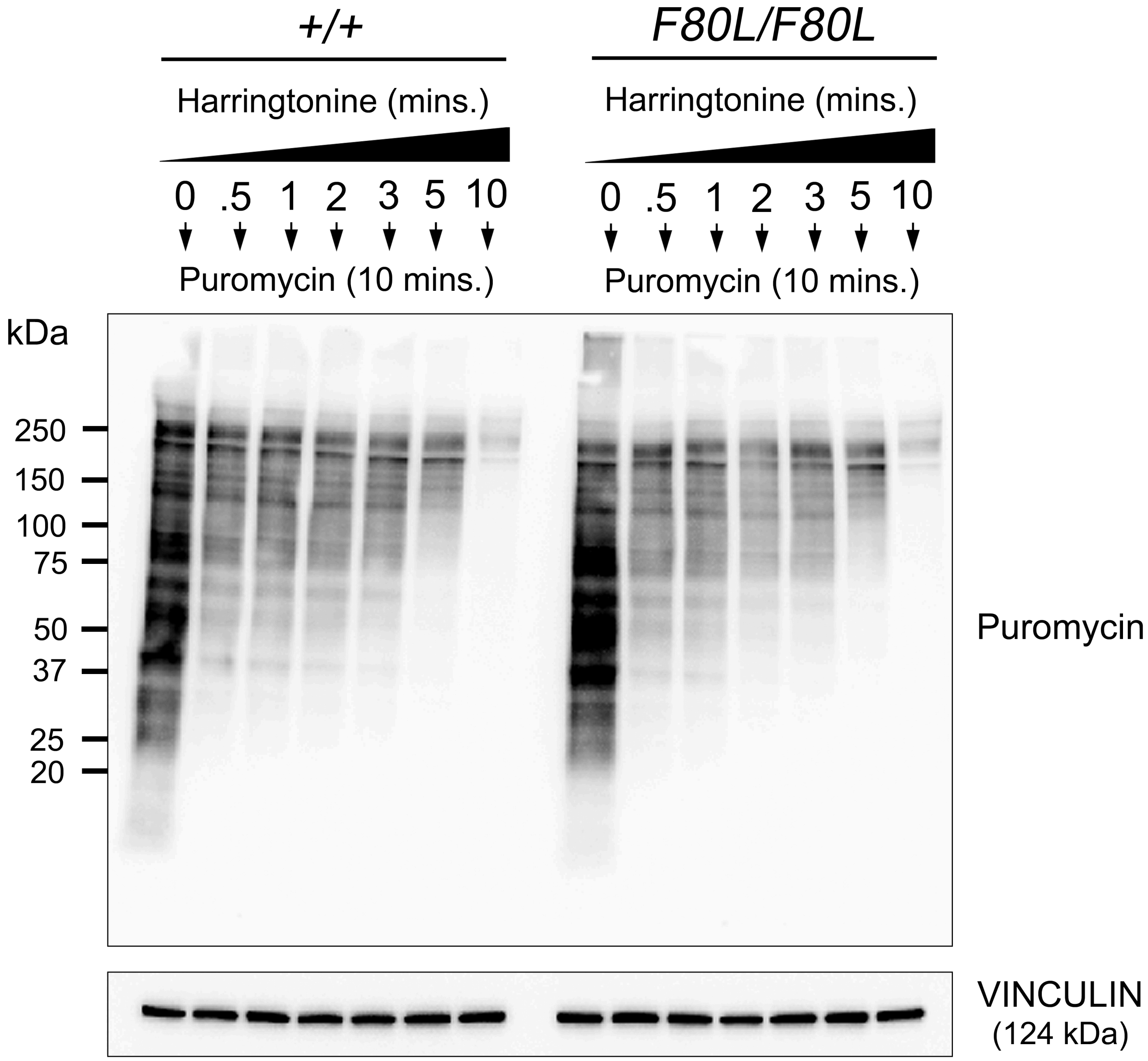

Supplementary Figure 8. SunRiSE (SUnSET-based Ribosome Speed of Elongation) analysis. Western blot analysis following the SunRiSE assay revealed no change in the rate of translation elongation between wild type and *Ronin*$^{F80L/F80L}$ MEFs. N = 3 biologically independent samples per genotype. Source data are provided as a Source Data file.

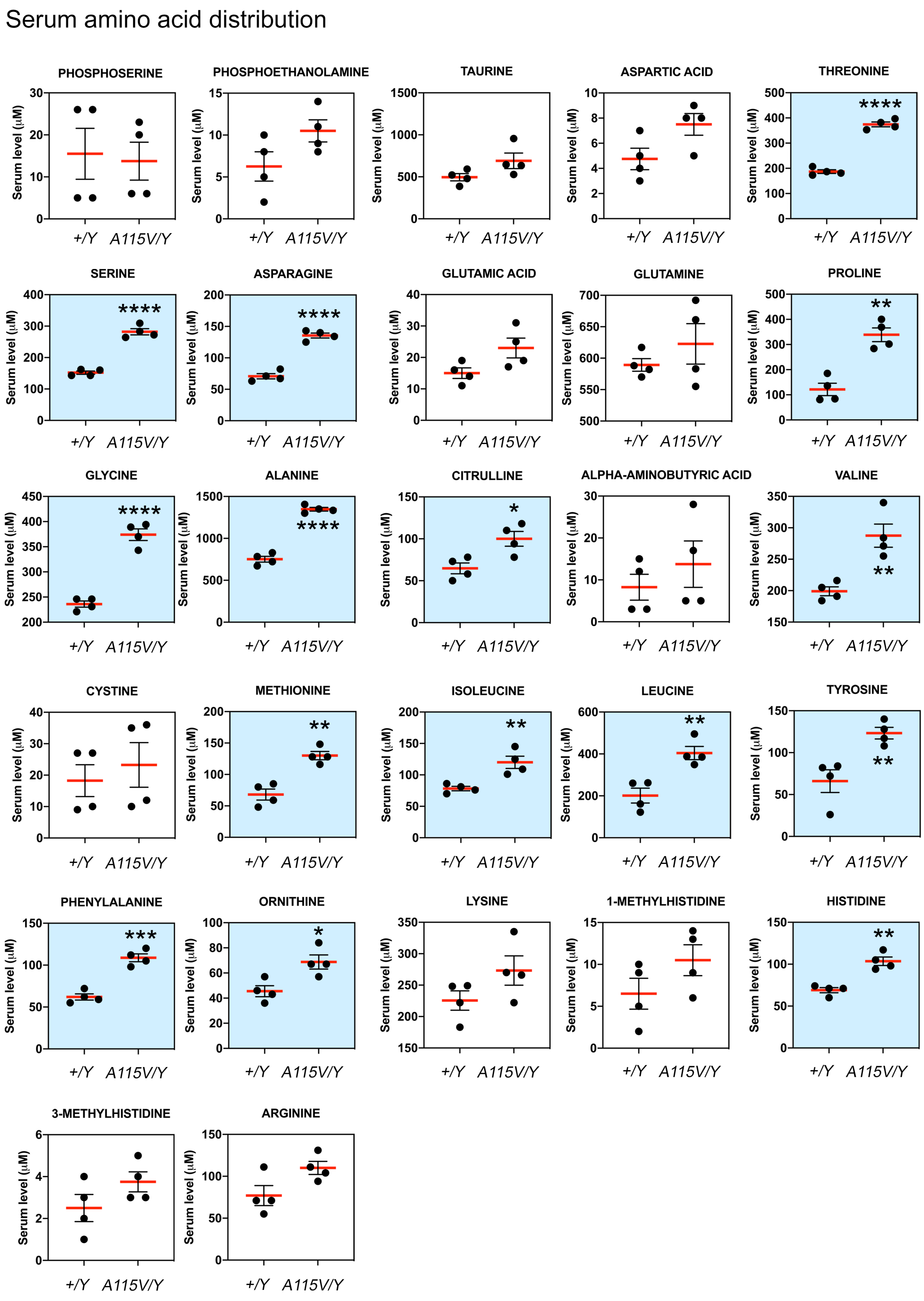

Supplementary Figure 9. Amino acid distribution in the *Hcfc1 A115V* mouse model.
Several amino acids (blue shaded) in *Hcfc1*$^{A115V/Y}$ adult plasma were increased as compared to wild type. Data shown a mean ± SEM. N = 4 biologically independent samples per genotype. Statistically significant differences between genotypes were determined using the *t* test (two-tailed). *p<0.05, **p<0.01, ***p<0.001, ****p<0.0001. Source data are provided as a Source Data file.

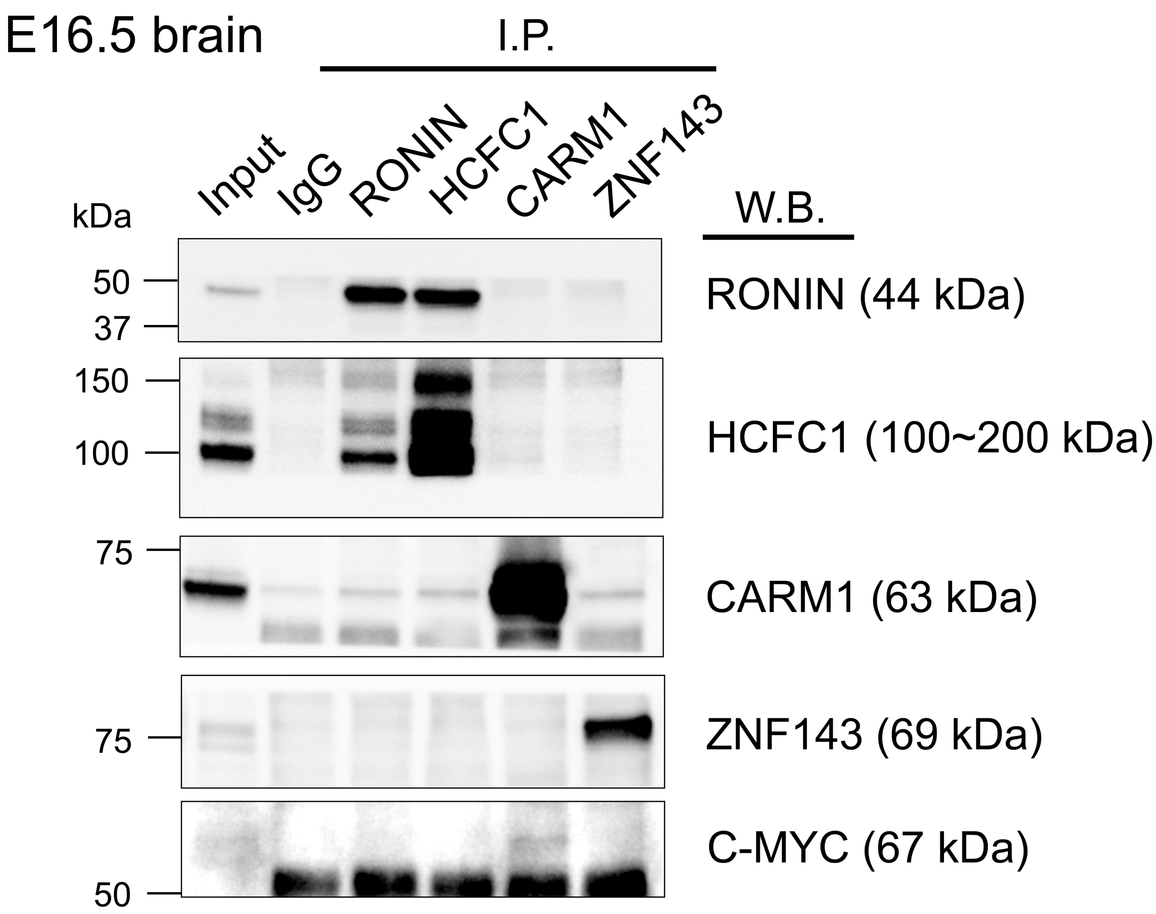

Supplementary Figure 10. RONIN and HCFC1 co-immunoprecipitation from E16.5 mouse brain lysates. Western blot analysis of RONIN and HCFC1 immunoprecipitation failed to detect interaction with ZNF143 or C-MYC. N = 3 biologically independent samples per genotype. Source data are provided as a Source Data file.

REFERNECES

1. Voiculescu, O., Charnay, P. & Schneider-Maunoury, S. Expression pattern of a Krox-20/Cre knock-in allele in the developing hindbrain, bones, and peripheral nervous system. *Genesis* **26**, 123-6 (2000).
2. Scott, M.M., Williams, K.W., Rossi, J., Lee, C.E. & Elmquist, J.K. Leptin receptor expression in hindbrain Glp-1 neurons regulates food intake and energy balance in mice. *J Clin Invest* **121**, 2413-21 (2011).
3. Dejosez, M. *et al.* Ronin/Hcf-1 binds to a hyperconserved enhancer element and regulates genes involved in the growth of embryonic stem cells. *Genes Dev* **24**, 1479-84 (2010).
4. Poche, R.A. *et al.* RONIN Is an Essential Transcriptional Regulator of Genes Required for Mitochondrial Function in the Developing Retina. *Cell Rep* **14**, 1684-1697 (2016).
