# Supplementary material for: Mutations in Hcfc1 and Ronin result in an inborn error of cobalamin metabolism and ribosomopathy
Source: Nat Commun. 2022 Jan 10;13:134. doi: 10.1038/s41467-021-27759-7 (PMC8748873; doi:10.1038/s41467-021-27759-7)
Supplement: Supplementary file 2 — Description of Additional Supplementary Files [file 41467_2021_27759_MOESM2_ESM.docx]

**Inventory of supplementary information**

**Supplementary Movie 1**. Movie of *Wild type* and *Ronin^F80L/F80L^* pups post-Caesarean section.

**Supplementary Dataset 1.** *Wild type* vs. *Ronin^F80L/F80L^* E16.5 brain RNA-seq and gene ontology

**Supplementary Dataset 2.** E16.5 brain RONIN ChIP-seq motif enrichment

**Supplementary Dataset 3.** E16.5 brain RONIN ChIP-seq peak annotation

**Supplementary Dataset 4.** E16.5 brain and P0 retina RONIN ChIP-seq peak overlap

**Supplementary Dataset 5.** P0 retina RONIN ChIP-seq unique peaks

**Supplementary Dataset 6.** E16.5 brain RONIN ChIP-seq unique peaks

**Supplementary Dataset 7.** E16.5 brain RONIN ChIP-seq unique peaks gene ontology

**Supplementary Dataset 8.** Overlay between E16.5 *Ronin^F80L/F80L^* brain RNA-seq (reduced transcripts) and RONIN ChIP-seq with gene ontology

**Supplementary Dataset 9.** Overlay between E16.5 *Ronin^F80L/F80L^* brain RNA-seq (increased transcripts) and RONIN ChIP-seq with gene ontology

**Supplementary Dataset 10.** *Wild type vs. Ronin^F80L/F80L^* mouse embryonic fibroblast mass spectrometry

**Supplementary Dataset 11.** Gene ontology of proteins enriched in *Ronin^F80L/F80L^ vs. wild type* mouse embryonic fibroblast mass spectrometry

**Supplementary Dataset 12.** Gene ontology of proteins reduced in *Ronin^F80L/F80L^ vs. wild type* mouse embryonic fibroblast mass spectrometry

**Supplementary Dataset 13.** *Wild type vs. Ronin^F80L/F80L^* E18.5 brain mass spectrometry

**Supplementary Dataset 14.** Gene ontology of proteins enriched in *Ronin^F80L/F80L^ vs. wild type* E18.5 brain mass spectrometry

**Supplementary Dataset 15.** Gene ontology of proteins reduced in *Ronin^F80L/F80L^ vs. wild type* E18.5 brain mass spectrometry
