# Supplementary material for: Mutations in Hcfc1 and Ronin result in an inborn error of cobalamin metabolism and ribosomopathy
Source: Nat Commun. 2022 Jan 10;13:134. doi: 10.1038/s41467-021-27759-7 (PMC8748873; doi:10.1038/s41467-021-27759-7)
Supplement: Supplementary file 4 — Reporting summary [file 41467_2021_27759_MOESM4_ESM.pdf]

nature research

Corresponding author(s): Ross A. Poche
NCOMMS-20-51280-T

Last updated by author(s): Nov. 19th, 2021

Reporting Summary

Nature Research wishes to improve the reproducibility of the work that we publish. This form provides structure for consistency and transparency in reporting. For further information on Nature Research policies, see our Editorial Policies and the Editorial Policy Checklist.

Statistics

For all statistical analyses, confirm that the following items are present in the figure legend, table legend, main text, or Methods section.

n/a	Confirmed	
☐	☒	The exact sample size (*n*) for each experimental group/condition, given as a discrete number and unit of measurement
☐	☒	A statement on whether measurements were taken from distinct samples or whether the same sample was measured repeatedly
☐	☒	The statistical test(s) used AND whether they are one- or two-sided<br>*Only common tests should be described solely by name; describe more complex techniques in the Methods section.*
☐	☒	A description of all covariates tested
☐	☒	A description of any assumptions or corrections, such as tests of normality and adjustment for multiple comparisons
☐	☒	A full description of the statistical parameters including central tendency (e.g. means) or other basic estimates (e.g. regression coefficient) AND variation (e.g. standard deviation) or associated estimates of uncertainty (e.g. confidence intervals)
☐	☒	For null hypothesis testing, the test statistic (e.g. *F*, *t*, *r*) with confidence intervals, effect sizes, degrees of freedom and *P* value noted<br>*Give P values as exact values whenever suitable.*
☒	☐	For Bayesian analysis, information on the choice of priors and Markov chain Monte Carlo settings
☒	☐	For hierarchical and complex designs, identification of the appropriate level for tests and full reporting of outcomes
☒	☐	Estimates of effect sizes (e.g. Cohen's *d*, Pearson's *r*), indicating how they were calculated

Our web collection on statistics for biologists contains articles on many of the points above.

Software and code

Policy information about availability of computer code

Data collection	StepOnePlus Real-Time PCR System (Life Technologies), EZchrome, Waters QuantiLynx, hematology analyzer (Scil Vet abc animal blood counter), Zeiss ZEN 2.3 (black), Zeiss ZEN (blue), Logger Lite (Vernier), Bruker SkyScan 1272 microCT system.
Data analysis	Graph Pad Prism, ImageJ, NRecon, CTAn, Imaris, Deformetrica, Image Lab (BioRad).<br>R packages for RNA-seq analysis: STAR (version 2.7.3a), DESeq2 (version 2.13), Metascape. For ChIP-seq analysis: bowtie2 (version 2.3.4.2), HOMER (version v4.10.3). For mass sepctrometry: Proteome Discoverer (version 2.0.0.802), Mascot search engine (v2.5.1), Percolator (v2.05), gpGrouper (v1.0.040), DAVID (v6.8). Landmark-free morphometrics analysis: Toussaint, N. et al. Application of high-resolution landmark-free morphometrics to a mouse model of Down Syndrome reveals a tightly localised cranial phenotype. bioRxiv (2020).The pipeline code is published on GitLab at https://gitlab.com/ntoussaint/landmark-free-morphometry.

For manuscripts utilizing custom algorithms or software that are central to the research but not yet described in published literature, software must be made available to editors and reviewers. We strongly encourage code deposition in a community repository (e.g. GitHub). See the Nature Research guidelines for submitting code & software for further information.

Data

Policy information about availability of data

All manuscripts must include a data availability statement. This statement should provide the following information, where applicable:

- Accession codes, unique identifiers, or web links for publicly available datasets
- A list of figures that have associated raw data
- A description of any restrictions on data availability

To review RNA-seq and ChIP-seq data (GEO accession GSE161763), go to https://www.ncbi.nlm.nih.gov/geo/query/acc.cgi?acc=GSE161763. Enter token

epijoagkbrulhov into the box

Mass spectrometry raw data files are deposited at ProteomeXchange (PDX0022310), ftp://massive.ucsd.edu/MSV000086402.

Field-specific reporting

Please select the one below that is the best fit for your research. If you are not sure, read the appropriate sections before making your selection.

- [x] Life sciences
- [ ] Behavioural & social sciences
- [ ] Ecological, evolutionary & environmental sciences

For a reference copy of the document with all sections, see nature.com/documents/nr-reporting-summary-flat.pdf

Life sciences study design

All studies must disclose on these points even when the disclosure is negative.

Sample size	Sample sizes were determined based on previously published studies in the field that employ similar experimental design as well as our own prior studies.
Data exclusions	No data were excluded from this study.
Replication	For all mouse experiments, independent cohorts, as well as biological replicates, were used. For cell line experiments, we used both biological and technical replicates. All attempts at replication were successful.
Randomization	Except for when specific genotypes were required, the mice were randomly assigned to different experimental groups.
Blinding	Prior to phenotyping, mass spec., ChIP-seq, and RNA-seq experiments, mouse samples were assigned unique identifiers to blind experimenters to genotypes.

Reporting for specific materials, systems and methods

We require information from authors about some types of materials, experimental systems and methods used in many studies. Here, indicate whether each material, system or method listed is relevant to your study. If you are not sure if a list item applies to your research, read the appropriate section before selecting a response.

Materials & experimental systems

n/a	Involved in the study	
☐	☒	Antibodies
☐	☒	Eukaryotic cell lines
☒	☐	Palaeontology and archaeology
☐	☒	Animals and other organisms
☒	☐	Human research participants
☒	☐	Clinical data
☒	☐	Dual use research of concern

Methods

n/a	Involved in the study	
☐	☒	ChIP-seq
☒	☐	Flow cytometry
☒	☐	MRI-based neuroimaging

Antibodies

Antibodies used	Western blot analysis (WB): RONIN (BD Biosciences, #562548), HCFC1 (Bethyl, #A301-400A), MMACHC (NeuroMabs, #N230/21), GFAP (DAKO, #Z0334), UBIQUITIN (Santa Cruz, #sc-8017), ETC Cocktail [ATP5A, UQCRC2, SDHB, NDUFB8, COX1] (Abcam, #ab110413), and ETC Native Blue Cocktail [NDUFA9, SDHA, UQCRC2, ATP5A, COX4] (Abcam, #ab110412), LETM1 (Proteintech, # 16024-1-AP), WDR12 (Abcam, #ab95070), Puromycin (Kerafast, #EQ0001), CARM1 (Bethyl, #A300-421A), C-MYC (Santa Cruz, #sc-40), and ZNF143 (Proteintech, #16618-1-AP), GAPDH (Millipore, #MAB374) and VINCULIN (Cell Signaling, #13901).<br><br>Co-immunoprecipitation (Co-IP): RONIN (Bethyl, #A303-180A), HCFC1 (Bethyl, #A301-400A), CARM1 (Bethyl, #A300-421A), C-MYC (Santa Cruz, #sc-40), and ZNF143 (Proteintech, #16618-1-AP), Rabbit IgG (Millipore, #12-370), Mouse IgG (Millipore, #12-371).<br><br>Chromatin immunoprecipitation (ChIP): RONIN (BD Biosciences, #562548), Mouse IgG (Millipore, #12-371).<br><br>Immunofluorescence (IF): GFAP (DAKO, #Z0334), AQP4 (Millipore, #AB2218), HuC/D (Invitrogen, #A-21271), Neurofilament (Millipore, #AB1987), alpha-BTX, Alexa Fluor 594 conjugate (Invitrogen, #B13423).<br><br>Secondary antibodies:<br>WB: goat, anti-Mouse IgG HRP Conjugate (Bio-Rad, #170-6516), goat, anti-Rabbit IgG HRP Conjugate (Bio-Rad, #170-6515). HRP-conjugated mouse anti-rabbit IgG (#211-032-171) and HRP-conjugated goat anti-mouse IgG (#115-035-174) both from Jackson Immuno Research Laboratories.

IF: goat, anti-Rabbit Alexa Fluor 488 (Invitrogen, # A-11034), goat, anti-mouse Alexa Fluor 555 (Invitrogen, # A-21424)

Validation

RONIN (BD Biosciences, #562548): WB, ChIP. Validated on manufactures' website:
https://www.bdbiosciences.com/eu/applications/research/stem-cell-research/pluripotent-stem-cell-markers-esc-and-ipsc/human/purified-mouse-anti-roninthap11-p56-507/p/562548

RONIN (Bethyl, #A303-180A): Co-IP. Validated on manufactures' website:
https://www.bethyl.com/product/A303-180A/Ronin+Antibody

HCFC1 (Bethyl, #A301-400A): WB, Co-IP. Validated on manufactures' website:
https://www.bethyl.com/product/A301-400A/HCF1+Antibody

MMACHC (NeuroMabs, #N230/21): WB. Validated on manufactures' website:
https://neuromab.ucdavis.edu/datasheet/N230_21.pdf

GFAP (DAKO, #Z0334): WB and IF. Validated by published papers:
Rueda and Hall et al. Cell Rep. 2019. PMID: 31067451

UBIQUITIN (Santa Cruz, #sc-8017): WB. Validated by published papers:
https://www.scbt.com/p/ubiquitin-antibody-a-5?gclid=EAIaIQobChMI1sTnkcqL7gIVkPDACh0zqgffEAAYASAAEgJ-6fD_BwE

ETC Cocktail (Abcam, #ab110413): WB. Validated by published papers:
https://www.abcam.com/total-oxphos-rodent-wb-antibody-cocktail-ab110413.html

ETC Native Blue Cocktail (Abcam, #ab110412): WB. Validated by published papers:
https://www.abcam.com/total-oxphos-blue-native-wb-antibody-cocktail-ab110412.html

LETM1 (Proteintech, # 16024-1-AP):WB. Validated on manufactures' website:
https://www.ptglab.com/products/LETM1-Antibody-16024-1-AP.htm

WDR12 (Abcam, #ab95070): WB. Validated on manufactures' website:
https://www.abcam.com/wdr12-antibody-ab95070.html

Puromycin (Kerafsat, #EQ0001): WB. Validated by published papers:
https://www.kerafast.com/productgroup/190/anti-puromycin-3rh11-antibody

CARM1 (Bethyl, #A300-421A): WB, Co-IP. Validated on manufactures' website:
https://www.bethyl.com/product/A300-421A/CARM1+Antibody

C-MYC (Santa Cruz, #sc-40): WB, Co-IP. Validated on manufactures' website and published papers:
https://www.scbt.com/p/c-myc-antibody-9e10
Das et al. Cell Death Differ. 2020.PMID: 32415280

ZNF143 (Proteintech, #16618-1-AP): WB, Co-IP. Validated on manufactures' website and published papers:
https://www.ptglab.com/products/ZNF143-Antibody-16618-1-AP.htm
Parker et al. Cell Rep. 2014. PMID: 25437553

GAPDH (Millipore, #MAB374): WB. Validated by published papers:
https://www.emdmillipore.com/US/en/product/Anti-Glyceraldehyde-3-Phosphate-Dehydrogenase-Antibody-clone-6C5,MM_NF-MAB374

VINCULIN (Cell Signaling, #13901): WB. Validated by published papers:
https://www.cellsignal.com/products/primary-antibodies/vinculin-e1e9v-xp-rabbit-mab/13901

AQP4 (Millipore, #AB2218): IF. Validated by published papers:
Yousef et al. Nat Med. 2019. PMID: 31086348

HuC/D (Invitrogen, #A-21271): IF. Validated on manufacturer's website:
https://www.thermofisher.com/antibody/product/HuC-HuD-Antibody-clone-16A11-Monoclonal/A-21271

Neurofilament (Millipore, #AB1987): IF. Validated on manufacturer's website:
https://www.emdmillipore.com/US/en/product/Anti-Neurofilament-M-145-kDa-Antibody-CT,MM_NF-AB1987

alpha-BTX, Alexa Fluor 594 conjugate (Invitrogen, #B13423): IF. Validated by published papers:
Makino et al. PLoS One. 2017. PMID: 29040265

Mouse IgG (Millipore, #12-371): Co-IP and ChIP . Validated on manufactures' website:
https://www.emdmillipore.com/US/en/product/Normal-Mouse-IgG,MM_NF-12-371

Rabbit IgG (Millipore, #12-370): Co-IP. Validated on manufactures' website:
https://www.emdmillipore.com/US/en/product/Normal-Rabbit-IgG,MM_NF-12-370

Secondary antibodies:
goat, anti-Mouse IgG HRP Conjugate (Bio-Rad, #170-6516): WB. Validated on manufactures' website and published papers:
https://www.bio-rad.com/en-us/sku/1706516-goat-anti-mouse-igg-h-l-hrp-conjugate?ID=1706516
Poché et al. Cell Rep. 2016. PMID: 26876175
Rueda and Hall et al. Cell Rep. 2019. PMID: 31067451

goat, anti-Rabbit IgG HRP Conjugate (Bio-Rad, #170-6515): WB. Validated on manufactures' website and published papers:
https://www.bio-rad.com/en-us/sku/1706515-goat-anti-rabbit-igg-h-l-hrp-conjugate?ID=1706515
Poché et al. Cell Rep. 2016. PMID: 26876175
Rueda and Hall et al. Cell Rep. 2019. PMID: 31067451

HRP-conjugated mouse anti-rabbit IgG (Jackson Immuno Research Laboratories, #211-032-171): WB. Validated on manufactures' website:
https://www.jacksonimmuno.com/catalog/products/211-032-171

HRP-conjugated goat anti-mouse IgG (Jackson Immuno Research Laboratories, #115-035-174): WB. Validated on manufactures' website:
https://www.jacksonimmuno.com/catalog/products/115-035-174

goat, anti-Rabbit Alexa Fluor 488 (Invitrogen, # A-11034): IF. Validated on manufactures' website:
https://www.thermofisher.com/antibody/product/Goat-anti-Rabbit-IgG-H-L-Highly-Cross-Adsorbed-Secondary-Antibody-Polyclonal/A-11034

goat, anti-mouse Alexa Fluor 555 (Invitrogen, # A-21424): IF. Validated on manufactures' website:
https://www.thermofisher.com/antibody/product/Goat-anti-Mouse-IgG-H-L-Highly-Cross-Adsorbed-Secondary-Antibody-Polyclonal/A-21424

Eukaryotic cell lines

Policy information about cell lines

Cell line source(s)	Mouse embryonic fibroblasts (MEFs) collected from Ronin F80L and Hcfc1 A115V mouse lines, O9-1 mouse neural crest cell line, HeLa cells, HEK293T cells.
Authentication	MEFs from both Ronin F80L and Hcfc1 A115V lines were authenticated based on the genotyping and gene expression patterns by qrtPCR and Western blotting.
Mycoplasma contamination	All cell lines were negative for mycoplasma contamination.
Commonly misidentified lines (See ICLAC register)	No commonly misidentified lines in this study.

Animals and other organisms

Policy information about studies involving animals; ARRIVE guidelines recommended for reporting animal research

Laboratory animals	The following mouse stains (Mus musculus) were used in this study: C57BL/6J, Ronin floxed, Wnt1-Cre2+/tg, Prrx1-Cre+/tg, Krox20-Cre+/tg, Phox2b-Cre+/tg, and Rpl24 Bst (from The Jackson Laboratory). Ronin F80L, Hcfc1 A115V, Mmachc floxed, Mmachc-OE+/tg lines were generated by our laboratory with the service of Baylor College of Medicine Mouse Embryonic Stem Cell Core.<br>Since there were no age- or sex-dependent differences in our observations, both male and female animals were used with the exception of Hcfc1 A115V hemizygous males compared to wild type males.
Wild animals	No wild animals were used
Field-collected samples	No field-collected samples were used
Ethics oversight	All animal research was conducted according to protocols approved by the Institutional Animal Care and Use Committee (IACUC) of Baylor College of Medicine.

Note that full information on the approval of the study protocol must also be provided in the manuscript.

ChIP-seq

Data deposition

☒ Confirm that both raw and final processed data have been deposited in a public database such as GEO.

☒ Confirm that you have deposited or provided access to graph files (e.g. BED files) for the called peaks.

Data access links<br>*May remain private before publication.*	To review GEO accession GSE161763:<br>Go to https://www.ncbi.nlm.nih.gov/geo/query/acc.cgi?acc=GSE161763<br>Enter token epijoagkbrulhov into the box.
Files in database submission	PEAK file, and bedgraph file
Genome browser session<br>(e.g. UCSC)	https://genome.ucsc.edu/s/mchill/Ronin_ChIPseq_InitialSubmission

Methodology

Replicates	Two biological replicates derived from different pools of embryonic brain tissue.
Sequencing depth	Each library was sequenced at a depth of ~ 90 million reads
Antibodies	RONIN (BD Biosciences, #562548), Mouse IgG (Millipore, #12-371).
Peak calling parameters	ChIP enriched peaks were identified using Homer with default transcription factor settings (findPeaks -style factor). All Ronin ChIP-seq peaks were called using input ChIP-seq data as background.
Data quality	Default settings for Homer findPeaks -style factor is an FDR threshold of 0.001 with at least a 4-fold more normalized tags than the input library. We identified 33,394 peaks with a peak score greater than 1.95.
Software	Reads were mapped to the mm9 assembly (NCBI Build 37) using bowtie2 (version 2.3.4.2). The ChIP-Seq peaks were called using HOMER (version v4.10.3). And the ChIP-seq signal was normalized to a 10 million reads total, and visualized in the UCSC genome browser after tag directories were generated using HOMER (version v4.10.3).
